# Neuronal-specific TNFAIP1 ablation attenuates postoperative cognitive dysfunction via targeting SNAP25 for K48-linked ubiquitination

**DOI:** 10.1186/s12964-023-01390-z

**Published:** 2023-12-15

**Authors:** Wei Wang, Wenwei Gao, Ping Gong, Wenqin Song, Xueshan Bu, Jiabao Hou, Lei Zhang, Bo Zhao

**Affiliations:** 1 Department of Anesthesiology, Renmin Hospital of Wuhan University, No.238 Jiefang Road, Wuhan, 430060 China; 2 Department of Critical Care Medicine, Renmin Hospital of Wuhan University, Wuhan, 430060 China; 3 State Key Laboratory of Oral & Maxillofacial Reconstruction and Regeneration, Key Laboratory of Oral Biomedicine Ministry of Education, Hubei Key Laboratory of Stomatology, Department of Anesthesiology, School & Hospital of Stomatology, Wuhan University, Wuhan, 430079 China

**Keywords:** TNFAIP1, SNAP25, Ubiquitination, Postoperative cognitive dysfunction, Mitophagy, Pyroptosis

## Abstract

**Background:**

Synaptosomal-associated protein 25 (SNAP25) exerts protective effects against postoperative cognitive dysfunction (POCD) by promoting PTEN-induced kinase 1 (PINK1)/Parkin-mediated mitophagy and repressing caspase-3/gasdermin E (GSDME)-mediated pyroptosis. However, the regulatory mechanisms of SNAP25 protein remain unclear.

**Methods:**

We employed recombinant adeno-associated virus 9 (AAV9)-hSyn to knockdown tumor necrosis factor α-induced protein 1 (TNFAIP1) or SNAP25 and investigate the role of TNFAIP1 in POCD. Cognitive performance, hippocampal injury, mitophagy, and pyroptosis were assessed. Co-immunoprecipitation (co-IP) and ubiquitination assays were conducted to elucidate the mechanisms by which TNFAIP1 stabilizes SNAP25.

**Results:**

Our results demonstrated that the ubiquitin ligase TNFAIP1 was upregulated in the hippocampus of mice following isoflurane (Iso) anesthesia and laparotomy. The N-terminal region (residues 1–96) of TNFAIP1 formed a conjugate with SNAP25, leading to lysine (K) 48-linked polyubiquitination of SNAP25 at K69. Silencing TNFAIP1 enhanced SH-SY5Y cell viability and conferred antioxidant, pro-mitophagy, and anti-pyroptosis properties in response to Iso and lipopolysaccharide (LPS) challenges. Conversely, TNFAIP1 overexpression reduced HT22 cell viability, increased reactive oxygen species (ROS) accumulation, impaired PINK1/Parkin-dependent mitophagy, and induced caspase-3/GSDME-dependent pyroptosis by suppressing SNAP25 expression. Neuron-specific knockdown of TNFAIP1 ameliorated POCD, restored mitophagy, and reduced pyroptosis, which was reversed by SNAP25 depletion.

**Conclusions:**

In summary, our findings demonstrated that inhibiting TNFAIP1-mediated degradation of SNAP25 might be a promising therapeutic approach for mitigating postoperative cognitive decline.

Video Abstract

**Supplementary Information:**

The online version contains supplementary material available at 10.1186/s12964-023-01390-z.

## Background

Postoperative cognitive dysfunction (POCD) refers to a cluster of cognitive disturbances persisting for weeks or months after anesthesia and surgery [1]. Geriatric patients are particularly susceptible to experiencing prolonged memory deterioration and impaired information processing, resulting in soaring economic burden due to limited daily activities and higher odds of mortality and dementia [2]. However, the underlying mechanisms of POCD remain unclear.

As a member of the soluble N-ethylmaleimide-sensitive factor attachment receptor (SNARE) complex, synaptosomal-associated protein 25 (SNAP25) plays a crucial role in modulating neurotransmitter release, vesicle trafficking, and synaptic plasticity [3]. Studies on SNAP25-deficient mice have shown reduced dendritic spine density and impaired long-term potentiation (LTP) in the hippocampus [4]. A restraint of SNAP25 protein was found in the hippocampus of rodents undergoing exploratory laparotomy under isoflurane (Iso) anesthesia [5, 6]. Our previous research demonstrated that SNAP25 mitigated surgery-induced cognitive impairment by enhancing PTEN-induced kinase 1 (PINK1)/Parkin-dependent mitophagy as well as repressing caspase-3/gasdermin E (GSDME)-dependent pyroptosis [5]. However, the regulatory mechanism by which surgery affects SNAP25 protein expression remains poorly understood.

Reversible post-translational modification with ubiquitin (Ub) is a crucial process involved in various dynamic cellular activities, including cell cycle transition, DNA repair, and signal transduction [7]. The conjugation of the 76-amino-acid (aa) ubiquitin to substrates entails E1 ubiquitin-activating enzymes, E2 ubiquitin-conjugating enzymes, and E3 ubiquitin ligases. The specificity of substrate recognition is determined by the ligases. Ubiquitin can be conjugated as a monomer to one or multiple lysine (K) residues within a substrate, termed monoubiquitination or multi-monoubiquitylation, respectively. Alternatively, ubiquitin can form chains utilizing seven lysine residues (K6, K11, K27, K29, K33, K48, and K63) and the N-terminal methionine (M1), resulting in polyubiquitination with distinct functional consequences. For instance, K48 chains are involved in protein stabilization and subsequent proteolysis by the 26S proteasome, while K63 chains participate in DNA repair and signal transduction [8, 9].

Tumor necrosis factor α-induced protein 1 (TNFAIP1), also known as BACURD2 or B12, was firstly identified as a protein induced by TNFα as well as lipopolysaccharide (LPS) in umbilical vein endothelial cells [10]. TNFAIP1 belongs to the potassium (K +) channel tetramerization domain (KCTD) protein family [11], and possesses a conserved Broad-complex, Tramtrack and Bric-à-brac (BTB) domain, which acts as a substrate-specific adaptor mediating the polyubiquitination and degradation of RhoA, RhoB, CSNK2B and KCTD10 [12–15]. TNFAIP1 upregulation was detected across the postmortem brain regions of Alzheimer’s disease (AD) cohorts and in the hippocampal neurons of APP/PS1 mice [16, 17], suggesting its potential involvement in the pathogenesis of cognitive impairment. Emerging evidence indicates that TNFAIP1 knockdown (KD) rescues the reduced expression of multiple synaptic plasticity-related proteins, such as cyclic AMP response element-binding protein (CREB), postsynaptic density protein 95 (PSD95), and synapsin-1 in N2a cells exposed to di-(2-ethylhexyl) phthalate or formaldehyde [18, 19].

In this study, we observed elevated TNFAIP1 levels in the hippocampus of mice subjected to isoflurane anesthesia and laparotomy. Functional investigations further revealed that neuron-specific inhibition of TNFAIP1 alleviated postoperative cognitive disorders by intensifying PINK1/Parkin-dependent mitophagy and suppressing caspase-3/GSDME-dependent pyroptosis. Mechanistically, TNFAIP1 catalyzed the K48-linked ubiquitination and degradation of SNAP25.

## Methods

### Cell culture and treatment

Cell lines from iCell Bioscience Inc (China) were cultured in medium supplemented with 10% fetal bovine serum (FBS; Gibco, USA) and 1% penicillin/streptomycin (P/S; Gibco, USA) at 37 ℃ with 5% CO_2_. HT22 murine hippocampal neurons and HEK293T cells were maintained in Dulbecco's modified Eagle's medium (DMEM; Hyclone, USA), while SH-SY5Y neuronal cells were grown in DMEM/F-12 (Hyclone, USA). To mimic the POCD environment, neurons were exposed to volatile anesthetics and LPS [20]. SH-SY5Y and HT22 cells were transfected with the indicated plasmids for 36 h. The plates were then exposed to 3% isoflurane with pure oxygen for 30 min in an anesthetic chamber. Additionally, LPS was added to the culture medium at a final concentration of 1 μg/mL for an additional 12 h, according to emerging evidence that this concentration could effectively inhibit mitophagy in HT22 neurons [21].

### Antibodies and reagents

The following antibodies were used for immunoprecipitation (IP) and immunoblot (IB) analysis: Flag (#66008–4-Ig, Proteintech, 1: 100 for IP, 1: 5,000 for IB), Myc (#2278, CST, 1: 50 for IP; #16286–1-AP, Proteintech, 1: 5,000 for IB), GST (#66001–2-Ig, Proteintech, 1:5,000 for IB), His (#66,005–1-Ig, Proteintech, 1:5,000 for IB), HA (#66006–2-Ig, Proteintech, 1: 1,000 for IB), TNFAIP1 (#sc-515765, Santa, 1:50 for IP; #15320–1-AP, Proteintech, 1: 500 for IB), SNAP25 (#sc-73044, Santa, 1:500 for IP; #ab41455, Abcam, 1: 2,000 for IB). The following antibodies were used only for western blot (WB) analysis: PINK1 (#23274–1-AP, Proteintech, 1: 1,000), Parkin (#14060–1-AP, Proteintech, 1: 1,000), LC3 (#14600–1-AP, Proteintech, 1: 1,000), p62 (#39,749, CST, 1: 2,000), caspase-3 (#AF6311, Affbiotech, 1:1,000), Cleaved caspase-3 (#AF7022, Affbiotech, 1: 500), GSDME (#DF9705, Affbiotech, 1: 1,000), N-GSDME (#AF4016, Affbiotech, 1: 500), GAPDH (ab181602, Abcam, 1: 10,000). Horseradish peroxidase (HRP)-conjugated secondary antibodies (1: 10,000) were purchased from ASPEN. Isoflurane was provided by Abbott Laboratories (Shanghai, China). LPS (#HY-D1056) and proteasome inhibitor MG132 (#HY-13259) were purchased from MedChemExpress (China). The protein synthesis inhibitor cycloheximide (CHX, S7418) was obtained from Selleck (Houston, USA).

### Plasmid and constructs

The pcDNA3.1 vector was used to construct Flag-tagged full-length (FL) TNFAIP1 and its truncations (1–96, 97–206, and 207–316) (Sino Biological, China) by cloning the respective cDNAs into the BmtI and KpnI sites. Myc-tagged wild-type (WT) SNAP25 and its lysine (K)-to-arginine (R) mutants (K69R, K184R, 189R, and K201R) (Sino Biological, China) were constructed by cloning the respective cDNAs into the BmtI and XhoI sites of the pcDNA3.1 vector. The pRK5-HA-Ub and its Lys48- and Lys63-only mutants were purchased from Fenghui Biotechnology (China). Small interfering RNAs (siRNAs) targeting human and mouse TNFAIP1 were synthesized by RiboBio (China) with the sequences 5'-GGUUGGGCAACAAGUAUGUTT-3' and 5'-CGTCGCATTCATGTCAAGCGCTATA-3', respectively. The SNAP25 short-hairpin RNA (shSNAP25) sequence was 5'-CAGAATCGCCAGATCGACAGGATCA-3'. Recombinant adeno-associated virus serotype 9 (AAV9) bearing short hairpin RNAs (shRNAs) against murine TNFAIP1 and SNAP25 mRNA containing human synapsin (hSyn) promoter were constructed by OBiO Biotechnology (China).

### Co-immunoprecipitation (co-IP) and immunoblotting (IB)

Following a rinse with pre-chilled phosphate-buffered saline (PBS), cells were lysed using IP lysis buffer (Beyotime, China). The resulting supernatant obtained after centrifugation was transferred to new Eppendorf (EP) tubes containing appropriate antibodies and protein A/G-conjugated magnetic beads (BIO-RAD, USA). Immunoprecipitates were then washed with IP wash buffer, and the purified eluates were subjected to western blotting (WB) using relevant antibodies.

For immunoblotting, proteins were extracted from cells and hippocampi using radioimmunoprecipitation assay (RIPA) buffer (ASPEN, China). The specimens were resolved by sodium dodecyl sulfate–polyacrylamide gel electrophoresis (SDS-PAGE, ASPEN, China) and transferred onto polyvinylidene difluoride (PVDF) membranes (Millipore, USA). Subsequently, the membranes were blocked with 5% skim milk and incubated with primary and secondary antibodies. Immunodetection was performed using an enhanced chemiluminescence (ECL) assay kit (ASPEN, China).

### GST pull-down

Recombinant GST-tagged TNFAIP1 and His-tagged SNAP25 were expressed in Escherichia coli BL21. The GST fusion proteins immobilized on glutathione-Sepharose 4B beads (GE Healthcare, USA) were incubated with His-SNAP25. The beads were then washed with GST binding buffer and analyzed by SDS-PAGE and WB.

### CHX chase assay

To assess SNAP25 protein stability, HT22 cells were transfected with either the FL or truncated forms of Flag-TNFAIP1 for 36 h and treated with 10 μg/mL CHX for 0, 3, and 6 h. Western blotting was performed to determine the SNAP25 protein content, with the basal levels of SNAP25 expression at 0 h adjusted to analogous levels for comparison.

### Real-time quantitative polymerase chain reaction (RT-qPCR)

SH-SY5Y cells were transfected with siRNAs targeting Homo sapiens TNFAIP1 for 36 h. Total RNA was isolated using TRIpure reagent (ELK, China), and reverse-transcribed into complementary DNA (cDNA) using EntiLink™ 1st Strand cDNA Synthesis Super Mix (ELK, China). RT-qPCR was performed using the EnTurbo™ SYBR Green PCR SuperMix kit. The primer sequences for TNFAIP1 were 5′-GAATTGATGGCTGAAGCAAAGTA-3′ (forward) and 5’- TAGGGATGTGATGATGGGGAT-3′ (reverse), while for GAPDH were 5′-TCAAGAAGGTGGTGAAGCAGG-3′ (forward) and 5′-TCAAAGGTGGAGGAGTGGGT-3′ (reverse). The fold change of TNFAIP1 was calculated using the 2^−ΔΔCt^ method.

### Cell viability and cell death

For cell viability detection, 10 μL of cell counting kit-8 (CCK-8) reagent (MedChemExpress, China) was added to each well and incubated for 4 h. Microplate reader (Molecular Devices, USA) was used to assess the optical density (OD) at 450 nm. Cell death detection was performed by incubating cell suspensions with 5 μL Annexin V-FITC and 5 μL propidium iodide (PI, Sungene, China) at room temperature in the dark. The samples were analyzed using a CytoFLEX flow cytometer (Beckman Coulter, USA).

### Measurement of oxidative stress

Intracellular reactive oxygen species (ROS) content was assessed using the 2′, 7′-dichlorodihydrofluorescein diacetate (DCFH-DA) probe following the manufacturer's instructions (Beyotime, China). Cells were incubated with DCFH-DA in serum-free medium and the DCFH-DA fluorescence intensity was detected using flow cytometry to define the degree of oxidative stress.

### Animals

Male C57BL/6 mice aged 12 months and weighing 28–32 g were obtained from Hubei Provincial Centers for Disease Control and Prevention. They were acclimatized for one week in a specific pathogen-free facility with controlled conditions of 22–25 ℃, 50% humidity, and a 12-h light/dark cycle. The mice had ad libitum access to food and water. All animal experimental protocols were approved by the Animal Ethics Committee of Renmin Hospital of Wuhan University.

### AAV9 transduction

Anesthetized mice were immobilized in a stereotaxic apparatus. AAVs were bilaterally injected into the hippocampus at a rate of 0.1 µL/min, with 1 µL per side. The injection coordinates were as follows: anterioposterior (AP) -2.0 mm, mediolateral (L) ± 2.3 mm, dorsoventral (DV) -2.0 mm [22]. The injection needle was left in place for 10 min and slowly withdrawn to avert liquid leakage. One mouse from each group was sacrificed after two weeks of microinjection. The accuracy of the injection site was confirmed by observing the distribution of enhanced green fluorescent protein (EGFP) under an upright laser scanning confocal microscope (Zeiss, Germany).

### POCD model

Aseptic laparotomy under isoflurane anesthesia was performed to induce POCD, following previously described methods [23, 24]. Briefly, mice were anesthetized using a transparent chamber (Yuyan Bio, China) prefilled with 1.5–3% isoflurane and pure oxygen. A 1.5-cm midline incision was made to penetrate the peritoneum. Approximately 5-cm of the small intestine was exteriorized and gently rubbed for 10 min using sterile gauze moistened with saline. The incision was closed using 4–0 Vicryl sutures (Ethicon, USA) in layers. The entire surgical procedure lasted around 30 min. Throughout anesthesia and surgery, the rectal temperature of the mice was maintained at 37 ± 0.5 ℃ using a heating pad. Postoperative analgesia was provided topically with compound lidocaine cream. The control mice received 100% oxygen without undergoing surgery or anesthesia.

### Open-field test (OFT)

On the third day after surgery, the OFT was conducted to evaluate the spontaneous locomotor activity of the mice, following a previously described protocol [25]. Each mouse was gently placed in the center of a cubic arena measuring 45 × 45 × 45 cm^3^ and allowed to move freely for 5 min. The SuperMaze tracking system (Xinruan, China) was utilized to record the overall distance traveled and the duration spent in the center area.

### Fear conditioning test (FCT)

Hippocampus-dependent memory was assessed using the contextual fear conditioning test 4–5 d after laparotomy. In the training phase, mice were habituated in the conditioning chamber for 5 min, followed by a 30-s tone (4.5 kHz, 60 dB) and then a 5-s footshock (0.5 mA). In the test phase, mice were placed in the same chamber the following day for 5 min to assess contextual memory retrieval. Freezing, defined as immobility lasting more than 2 s excluding respiration, was automatically recorded using the Super Fear Conditioning Analysis System (Xinruan, China).

### Hematoxylin–eosin (H&E) staining

Twenty-four hours after behavior tests, the mice were sacrificed under deep anesthesia and the hippocampal tissues were removed rapidly. Hippocampal slices were fixed in 4% paraformaldehyde and dehydrated with gradient ethanol. The specimens were embedded by paraffin and swiftly sliced into Sects. (2–3 μm). Dewaxed sections were stained with H&E complying with the protocol provided by the manufacturer. Histopathological alterations of hippocampal neurons were observed under a 400 × upright microscope (OLYMPUS, Japan) (*n* = 3 per group).

### Terminal deoxynucleotidyl transferase mediated dUTP nick end labeling (TUNEL) assay

Cell death in the mouse hippocampi was assessed using a one-step TUNEL assay kit (Beyotime, China). Dewaxed hippocampal samples were incubated with Cyanine 3-labeled dUTP and terminal deoxynucleotidyl transferase (TdT) for 1.5 h at 37 ℃ in the dark, followed by three washes with PBS. Finally, the tissues were dyed with 4',6-diamidino-2-phenylindole (DAPI; Sigma, USA) and incubated for 10 min at room temperature in the dark. TUNEL-positive cells in the bilateral hippocampi were observed using an upright laser scanning confocal microscope (Zeiss, Germany) (*n* = 3 per group).

### Enzyme-linked immunosorbent assay (ELISA)

Blood samples were collected using sterilized EP tubes, and allowed to naturally coagulate at room temperature for 30 min. The supernatant was obtained by centrifugation at 2,000 g for 10 min and stored at -80 ℃. Hippocampal homogenates were centrifuged to obtain the supernatant. Meanwhile, the homogenates were tested for protein concentration using Bicinchoninic acid (BCA) assay (ASPEN, China) to determine the levels of cytokines per mg of protein. IL-1β and IL-18 concentrations in the serum and hippocampus were quantified using ELISA kits (ELK, China) following the manufacturer's instructions (*n* = 4 per group).

### Statistical analyses

Data were presented as mean ± standard error of the mean (SEM) and analyzed using an unpaired Student's *t*-test or one-way analysis of variance (ANOVA) followed by Tukey's post hoc test. GraphPad Prism version 9.0 (GraphPad Software Inc., USA) was used for statistical analysis. Statistical significance was defined as *P* < 0.05.

## Results

### TNFAIP1 directly bound to SNAP25

**Fig. 1:**
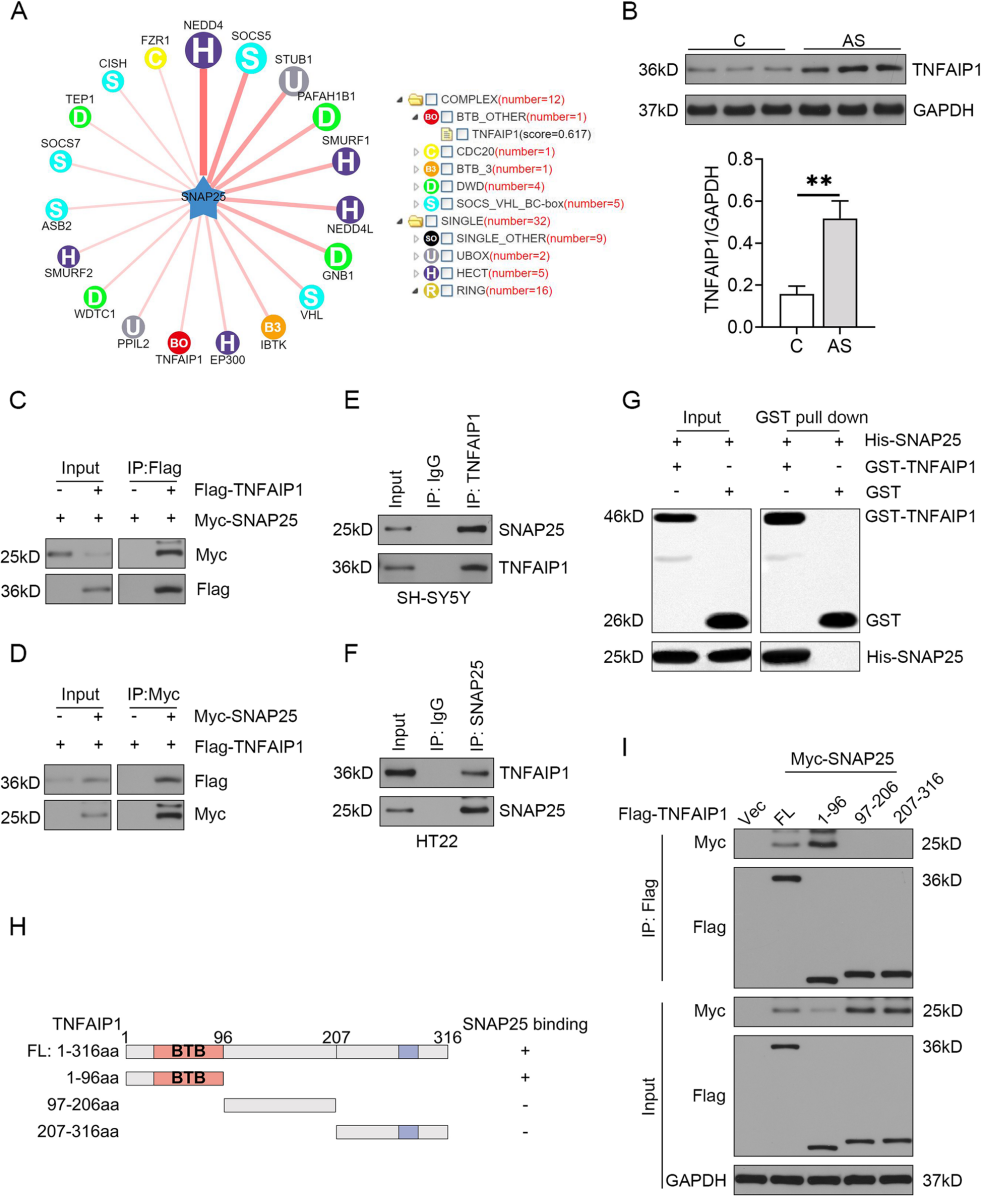
Identification of TNFAIP1 as an interacting protein with SNAP25. **A** Network view of E3–SNAP25 interactions (left panel) and the E3 hierarchical tree for SNAP25 (right panel). The predicted E3 ligases surrounding SNAP25 are represented different colors and characters according to their respective ubiquitin ligase domains. The size of the nodes, width of the edges, and shade of the edges indicate the confidence score. The position of the predicted ubiquitin ligases in the E3 family hierarchical tree is displayed. The abbreviations in each circle, such as "BO", "C" and "H", denote the E3 family, and the numbers in parentheses following each ubiquitin ligase family denote the number of corresponding predicted E3-SNAP25 interactions. **B** Western blot analysis of TNFAIP1 protein levels in the hippocampus of mice undergoing anesthesia and surgery (AS). Quantification results normalized to GAPDH are presented as mean ± SEM (*n* = 6). ^**^P < 0.01, unpaired *t*-test. **C, D** Co-IP using anti-Flag or anti-Myc antibody confirmed the interaction between exogenous TNFAIP1 and SNAP25 in HEK293T cells transfected with plasmids expressing Flag-TNFAIP1 and Myc-SNAP25. **E** Co-IP with an anti-TNFAIP1 antibody in SH-SY5Y cells confirmed the interaction between endogenous TNFAIP1 and SNAP25. **F** Co-IP with an anti-SNAP25 antibody in HT22 cells confirmed the interaction between endogenous TNFAIP1 and SNAP25. **G** TNFAIP1 directly interacted with SNAP25 in vitro. Purified GST (control) or GST-TNFAIP1 were mixed with His-SNAP25 and subjected to GST pull-down followed by IB for His. **H** Diagram illustrating the full-length and different truncations of TNFAIP1. Plus indicates binding, minus indicates no binding, and aa indicates amino acids. **I** Mapping of interacting domains of TNFAIP1 and SNAP25 by Co-IP with an anti-Flag antibody, followed by IB with anti-Flag or anti-Myc antibody

To determine the mechanism of SNAP25 downregulation in the progression of POCD, we utilized the publicly available database UbiBrowser [26] to predict the E3 ubiquitin ligase of SNAP25. A total of 44 putative E3 ubiquitin ligases that mediated the ubiquitination of SNAP25 were identified and depicted in Fig. 1A and Table S1. Due to its neuronal specificity [27] and involvement in neuroinflammation [28], TNFAIP1 was deemed a likely candidate, with a relative likelihood ratio of 3.01. Immunoblot analysis of the hippocampus of mice treated with anesthesia and surgery revealed increased levels of TNFAIP1 (Fig. 1B), leading us to hypothesize that TNFAIP1 may be a negative regulator of SNAP25. Coimmunoprecipitation assays confirmed the exogenous interaction between TNFAIP1 and SNAP25 (Fig. 1C, D). Additionally, the endogenous TNFAIP1 and SNAP25 were found to interact with each other in SH-SY5Y and HT22 cells (Fig. 1E, F). In vitro pull-down assays using GST-TNFAIP1 purified from Escherichia coli and immunopurified His-SNAP25 verified the direct interaction between TNFAIP1 and SNAP25, as evidenced by the presence of SNAP25 in fractions eluted from the GST-TNFAIP1 affinity column (Fig. 1G). To determine which domain of TNFAIP1 was responsible for binding to SNAP25, we introduced the FL TNFAIP1 or its three truncations (Fig. 1H) into HEK293T cells. Co-IP analysis revealed that the region spanning residues 1–96 of TNFAIP1, containing the BTB domain, was essential for binding to SNAP25 (Fig. 1I).

### TNFAIP1 ubiquitylated SNAP25 for degradation

**Fig. 2:**
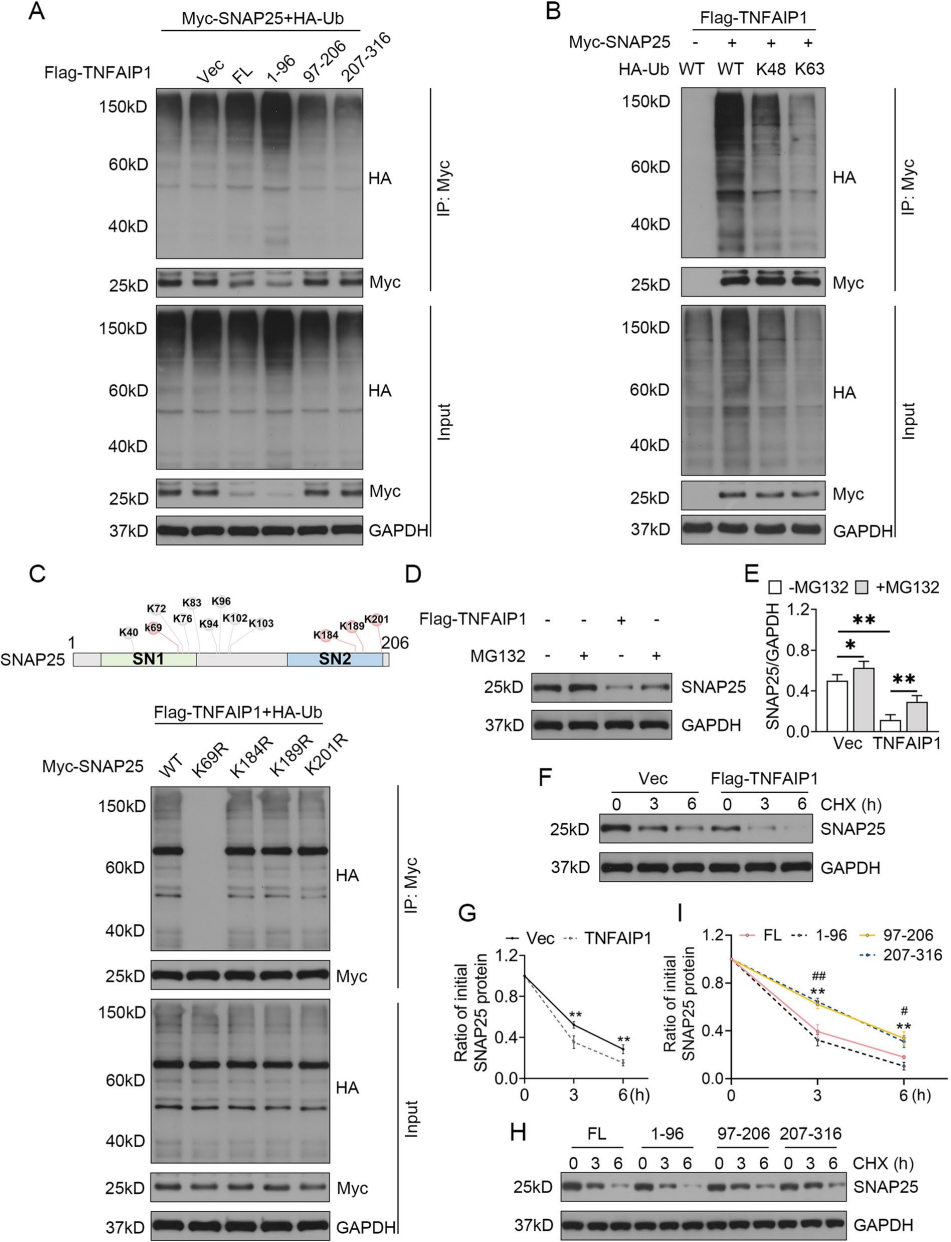
TNFAIP1 catalyzes K48-linked polyubiquitination of SNAP25 at K69. **A** Ubiquitination analysis of SNAP25 in lysates from HEK293T cells transfected with Myc-SNAP25, HA-Ub, and full-length Flag-tagged TNFAIP1 or its truncations. **B** Co-transfection of Flag-TNFAIP1 with Myc-SNAP25 and WT or mutant HA-tagged ubiquitin in HEK293T cells followed by ubiquitination assays of exogenous SNAP25. **C** Diagram illustrating the predicted ubiquitination sites of SNAP25 (colored in pink, top), and ubiquitination assays of SNAP25 in lysates from HEK293T cells transfected with Flag-TNFAIP1, HA-Ub, and WT or mutant Myc-tagged SNAP25 (bottom). **D** Western blot analysis of SNAP25 protein levels in HT22 cells treated with MG132 (50 nM, 4 h) and Flag-TNFAIP1. **E** Quantification results normalized to GAPDH are presented as mean ± SEM (*n* = 4). ^*^P < 0.05, ^**^P < 0.01, one-way ANOVA test. **F** Immunoblot analysis of SNAP25 in HT22 cells transfected with Flag-TNFAIP1 in the absence or presence of CHX (10 μg/mL). **G** The relative SNAP25 level for each time point was quantified as mean ± SEM (*n* = 3). ^**^P < 0.01, two-way ANOVA test. **H** HT22 cells transiently transfected with full-length Flag-tagged TNFAIP1 or its truncations were treated with 10 μg/mL CHX for 0, 3, and 6 h. **(I)** Quantification of SNAP25 levels relative to GAPDH was shown as mean ± SEM (*n* = 3). ^**^P < 0.01, 97–216 vs. FL; ^#^*P* < 0.05, ^##^*P* < 0.01, 207–316 vs. FL, two-way ANOVA test

The elevated ubiquitination level of SNAP25 was observed in HEK293T cells upon TNFAIP1 overexpression. Interestingly, the N-terminus of TNFAIP1 containing the BTB domain (residues 1–96) still facilitated SNAP25 ubiquitination as effectively as the FL protein, whereas the residues 97–206 and 207–316 of TNFAIP1 nearly completely abolished SNAP25 ubiquitination (Fig. 2A). Given that K48- and K63 linkages are the main types of polyubiquitin chains [29], we co-transfected HA-tagged Ub WT or Ub plasmids harboring only K48 or K63 residues with TNFAIP1 and SNAP25. We found that TNFAIP1 participated in the elongation of K48-specific polyubiquitin chains, but failed to augment the polyubiquitination of SNAP25 when all lysines of ubiquitin were mutated to arginines except K63 (Fig. 2B). Bioinformatics analysis of SNAP25 using the CKSAAP_UbSite webserver [30, 31] predicted four potential Ub-conjugation consensus motifs (Fig. 2C-top panel and Fig. S1A-B). To confirm these predictions, we constructed SNAP25 mutants bearing K to R substitutions in the predicted ubiquitination sites and performed ubiquitination assays. K69 was identified as a pivotal ubiquitination site of SNAP25, as reflected by the absence of ubiquitylation only when the K69R SNAP25 mutant was transduced into HEK293T cells (Fig. 2C-bottom panel). Proteins labeled with Lys48-linked ubiquitin chains are recognized by the proteasome that executes substrate degradation [32]. Western blot analysis demonstrated that TNFAIP1 overexpression in HT22 cells reduced SNAP25 protein levels. However, treatment with the proteasome inhibitor (MG132) prevented TNFAIP1-dependent SNAP25 degradation (Fig. 2D, E). Cycloheximide chase assay showed a shortened half-life of SNAP25 in HT22 cells with TNFAIP1 overexpression compared to the control group transfected with an empty vector (Fig. 2F, G). In addition, the C terminal truncations of TNFAIP1 (residues 97–206 and 207–316) prominently enhanced the SNAP25 protein stability compared to the full-length of TNFAIP1 (Fig. 2H, I). Taken together, our findings unveil that SNAP25 is a novel substrate of TNFAIP1 that mediates its ubiquitin-dependent proteasomal degradation at Lys 69.

### TNFAIP1 loss-of-function promoted PINK1/Parkin-dependent mitophagy and impeded caspase-3/GSDME-dependent pyroptosis in vitro

**Fig. 3:**
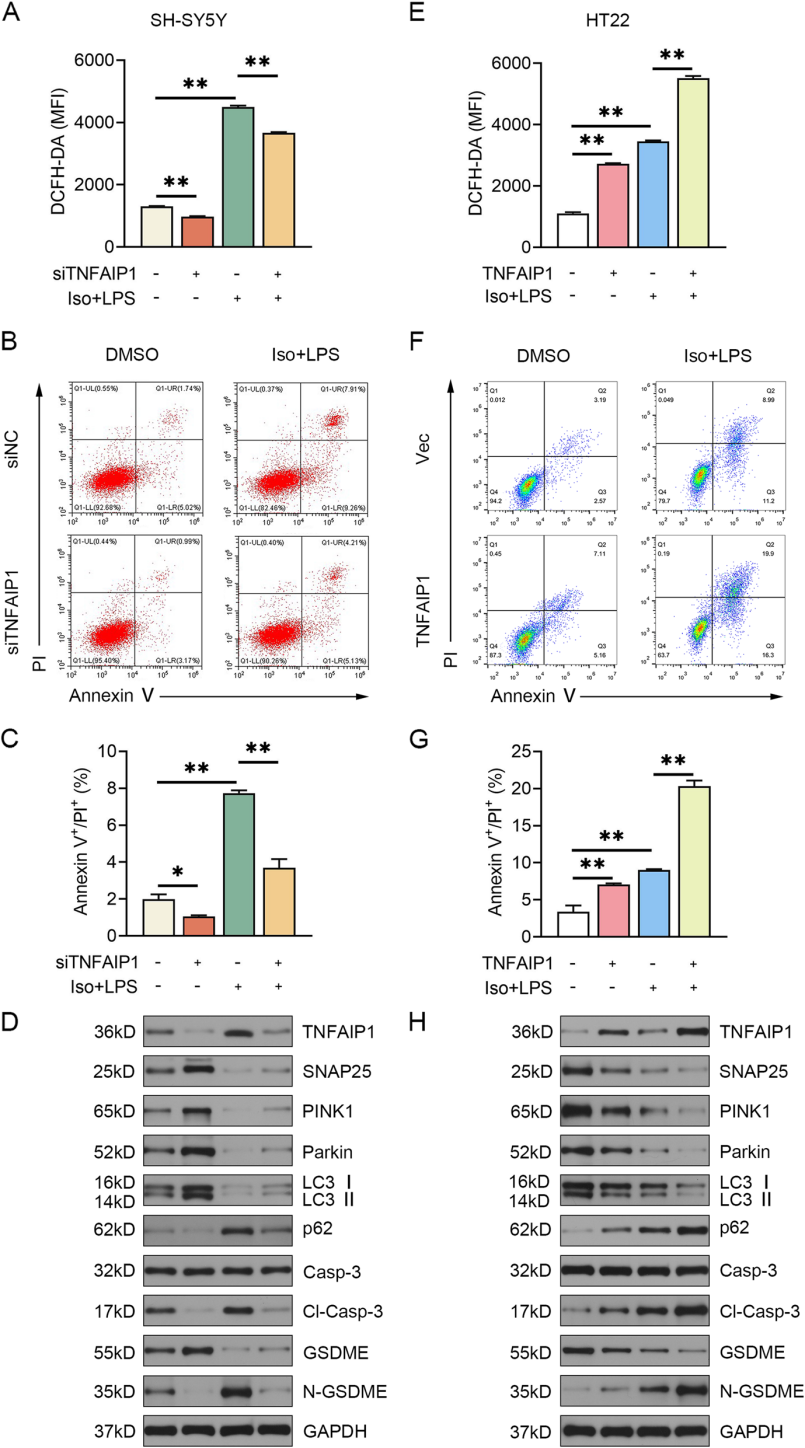
TNFAIP1 affects mitophagy and pyroptosis in vitro. **A** SH-SY5Y cells expressing control siRNA or siTNFAIP1 were treated with 3% isoflurane (30 min) and 1 μg/mL LPS (12 h), followed by incubation with 10 mM DCFH-DA for 20 min. The mean fluorescence intensity (MFI) of DCFH-DA was measured using flow cytometry to reflect the intracellular ROS level (*n* = 3). ^**^P < 0.01, one-way ANOVA test. **B** Flow cytometry analysis of cell death in SH-SY5Y cells using Annexin V-FITC/PI double-staining. **C** Quantification of pyroptotic cells with annexin V-FITC/PI double-positive staining (*n* = 3). ^*^P < 0.05, ^**^P < 0.01, one-way ANOVA test. **D** Gel images of mitophagy- and pyroptosis-related proteins in TNFAIP1-silenced SH-SY5Y cells. GAPDH served as a loading control. **E** HT22 cells expressing empty vector or TNFAIP1 were exposed to 3% isoflurane (30 min) and 1 μg/mL LPS (12 h), followed by incubation with 10 mM DCFH-DA for 20 min. The intracellular ROS level was measured by the MFI of DCFH-DA (*n* = 3). ^**^P < 0.01, one-way ANOVA test. **F** Flow cytometry analysis of double staining with Annexin V-FITC/PI in HT22 cells overexpressing TNFAIP1 after Iso + LPS treatment. **G** Quantification of pyroptotic cells with annexin V-FITC/PI double-positive staining (*n* = 3). ^**^P < 0.01, one-way ANOVA test. **H** Gel images of mitophagy- and pyroptosis-related proteins in TNFAIP1-overexpressed HT22 cells. GAPDH served as a loading control

The impaired crosstalk between PINK1/Parkin-mediated mitophagy and caspase-3/GSDME-mediated pyroptosis contributes to the progression and pathogenesis of POCD [33]. SNAP25 has been shown to confer postoperative cognitive benefits by facilitating mitophagy and hampering pyroptosis [5]. Accordingly, we hypothesized that TNFAIP1 may act in PINK1/Parkin-dependent mitophagy as well as caspase-3/GSDME-dependent pyroptosis via negatively regulating SNAP25. RT-qPCR results indicated that siTNFAIP1#1 was the most potent silencing fragment (Fig. S2A). The relative cell viability of SH-SY5Y cells significantly decreased upon Iso + LPS challenge, which was reversed by TNFAIP1 depletion (Fig. S2B). Increased fluorescence intensity of DCFH-DA, an indicator of intracellular ROS levels, was detected in Iso + LPS-treated SH-SY5Y cells. Nevertheless, TNFAIP1 silencing could scavenge intracellular ROS under Iso + LPS conditions, as indicated by weaker DCFH-DA fluorescence compared to the corresponding control cells (Fig. 3A). Exposure to Iso + LPS considerably increased the ratio of Annexin V/PI double-positive cells, which was abrogated by TNFAIP1 knockdown (Fig. 3B, C). WB analysis showed that isoflurane and lipopolysaccharide dramatically induced TNFAIP1 expression, but diminished SNAP25 expression in SH-SY5Y cells. Elevated SNAP25 expression was noted in TNFAIP1-deficient cells regardless of Iso + LPS treatment. Furthermore, TNFAIP1 knockdown markedly intensified PINK1/Parkin-driven mitophagy and repressed the cleavage of caspase-3 and GSDME in response to Iso + LPS stimulation (Fig. 3D and Fig. S3A-H). These findings highlight that TNFAIP1 ablation possesses neuroprotective properties by boosting PINK1/Parkin-dependent mitophagy and hindering caspase-3/GSDME-dependent pyroptosis.

### TNFAIP1 gain-of-function restrained PINK1/Parkin-dependent mitophagy and promoted caspase-3/GSDME-dependent pyroptosis in vitro

HT22 cells were transiently transfected with a plasmid encoding TNFAIP1 overexpression (Fig. S4A) to further illuminate the role of TNFAIP1 in neuronal mitophagy and pyroptosis. The relative cell viability of HT22 cells was significantly reduced after Iso + LPS treatment, which was further aggravated by TNFAIP1 overexpression (Fig. S4B). Excessive ROS production was also found in Iso + LPS-primed HT22 cells, which was further augmented by TNFAIP1 overexpression (Fig. 3E). The percentage of Annexin V/PI double-positive neurons was also significantly increased following Iso + LPS administration, and TNFAIP1 upregulation further augmented the number of pyroptotic cells (Fig. 3F, G). Iso + LPS-treated HT22 cells exhibited increased expression of TNFAIP1, p62, cleaved caspase-3, and N-GSDME, but decreased expression of SNAP25, PINK1, Parkin, and LC3-II. Notably, TNFAIP1 overexpression led to a substantial reduction in SNAP25 protein content regardless of Iso + LPS intervention. Further restraint of PINK1, Parkin, and LC3-II expression was observed in TNFAIP1-OE cells exposed to Iso + LPS, accompanied by more pronounced p62 accumulation. TNFAIP1 facilitated the cleavage of both caspase-3 and GSDME more easily compared to the empty vector (Fig. 3F, G and Fig. S5A-H). These results suggest that TNFAIP1 is responsible for Iso + LPS-provoked insufficient mitophagy and pyroptosis activation.

### TNFAIP1 triggered compromised mitophagy and excessive pyroptosis through suppressing SNAP25 expression

**Fig. 4:**
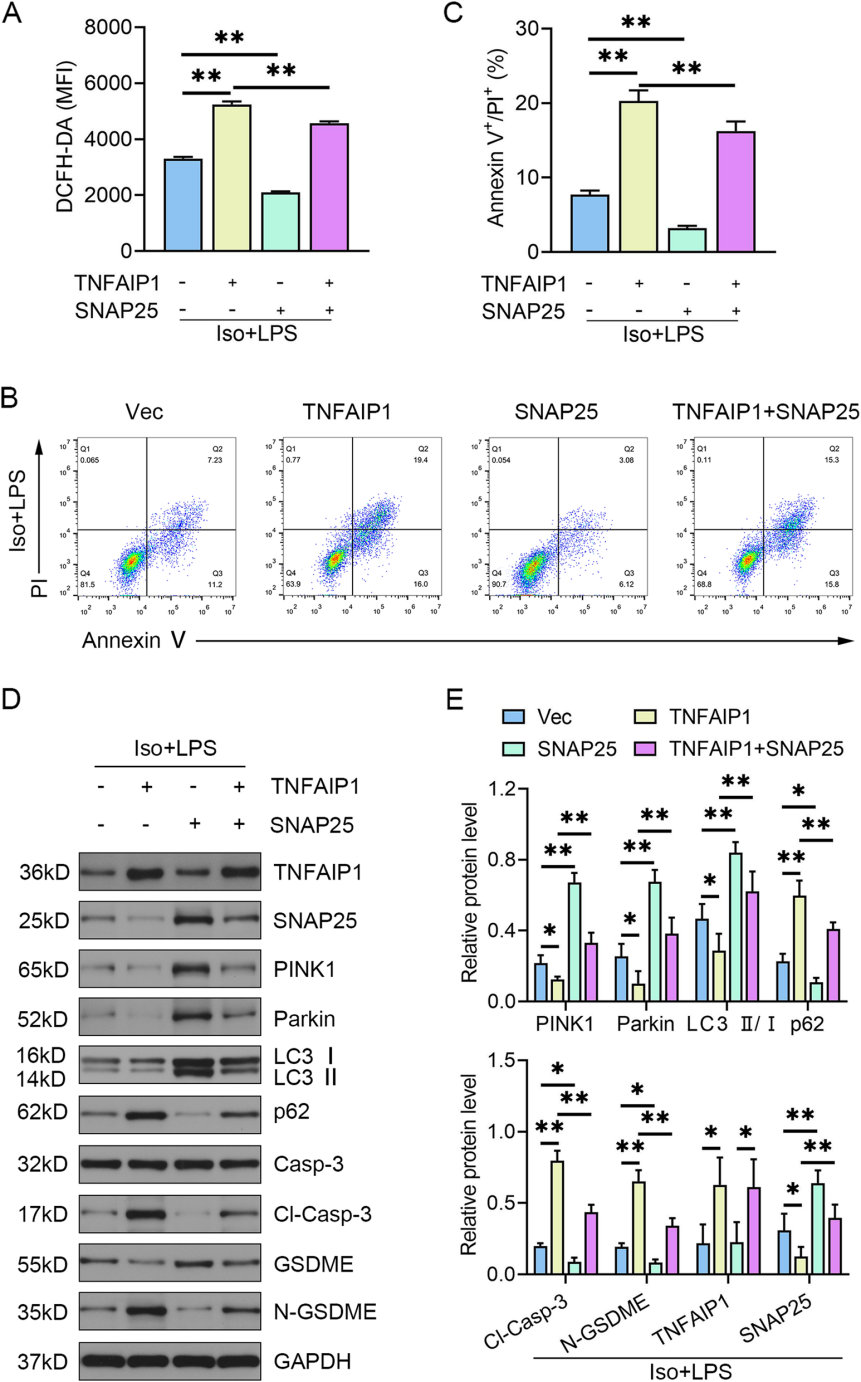
SNAP25 overexpression rescues the defective mitophagy and excessive pyroptosis phenotypes induced by TNFAIP1 overexpression. **A** HT22 cells were transfected with indicated plasmids and treated with 3% isoflurane (30 min) and 1 μg/mL LPS (12 h), followed by incubation with 10 mM DCFH-DA for 20 min. The intracellular ROS level was reflected by the MFI of DCFH-DA (*n* = 3). ^**^P < 0.01, one-way ANOVA test. **B** Flow cytometry analysis of double staining with Annexin V-FITC/PI under Iso + LPS conditions in HT22 cells overexpressing indicated plasmids. **C** Quantification of pyroptotic cells with annexin V-FITC/PI double-positive staining (*n* = 3). ^**^P < 0.01, one-way ANOVA test. **D** Gel images of mitophagy- and pyroptosis-related proteins in Iso + LPS-treated HT22 cells transfected with indicated plasmids. GAPDH served as a loading control. **E** Quantification results normalized to GAPDH were presented as mean ± SEM (*n* = 4). ^*^P < 0.05, ^**^P < 0.01, one-way ANOVA test

The Myc-SNAP25 plasmid (Fig. S6A) was introduced into HT22 cells in the presence or absence of TNFAIP1 overexpression to ascertain whether SNAP25 is necessary for the mitophagy and pyroptosis phenotypes governed by TNFAIP1. The reduced cell viability of HT22 cells caused by Iso + LPS challenge was reversed by Myc-SNAP25. Intriguingly, SNAP25 overexpression rescued the decrease in cell viability induced by TNFAIP1 overexpression (Fig. S6B). Myc-SNAP25 exhibited potent antioxidant properties in response to Iso + LPS stimulation, as evidenced by decreased fluorescence intensity of DCFH-DA in contrast with the vector group. Moreover, TNFAIP1/SNAP25 double overexpression cells showed lower levels of ROS generation following Iso + LPS exposure compared to TNFAIP1 overexpression alone (Fig. 4A). Pre-treatment with the Myc-SNAP25 plasmid prevented HT22 cells from undergoing pyroptosis upon Iso + LPS stimulation, as indicated by a reduced proportion of Annexin V/PI double-positive neurons. Concurrent overexpression of TNFAIP1 and SNAP25 decreased the ratio of Annexin V/PI double-stained cells in contrast with the TNFAIP1-OE treatment under Iso + LPS conditions (Fig. 4B, C). Immunoblot data demonstrated that SNAP25 overexpression rescued defective PINK1/Parkin-mediated mitophagy, facilitated the processing of LC3-I to LC3-II, inhibited abnormal p62 accumulation, and suppressed the activation of the caspase-3/GSDME axis. Additionally, upregulation of PINK1/Parkin signaling, enhanced conversion from LC3-I to LC3-II, along with diminished levels of p62, cleaved caspase-3, and N-GSDME were observed in neurons expressing both TNFAIP1 and SNAP25 compared to the TNFAIP1 group following Iso + LPS stimulation (Fig. 4D, E). Overall, our data illustrate that SNAP25 is involved in the insufficient mitophagy and pyroptosis activation phenotypes dominated by TNFAIP1.

### Selective neuronal ablation of TNFAIP1 alleviated POCD by augmenting SNAP25 expression

**Fig. 5:**
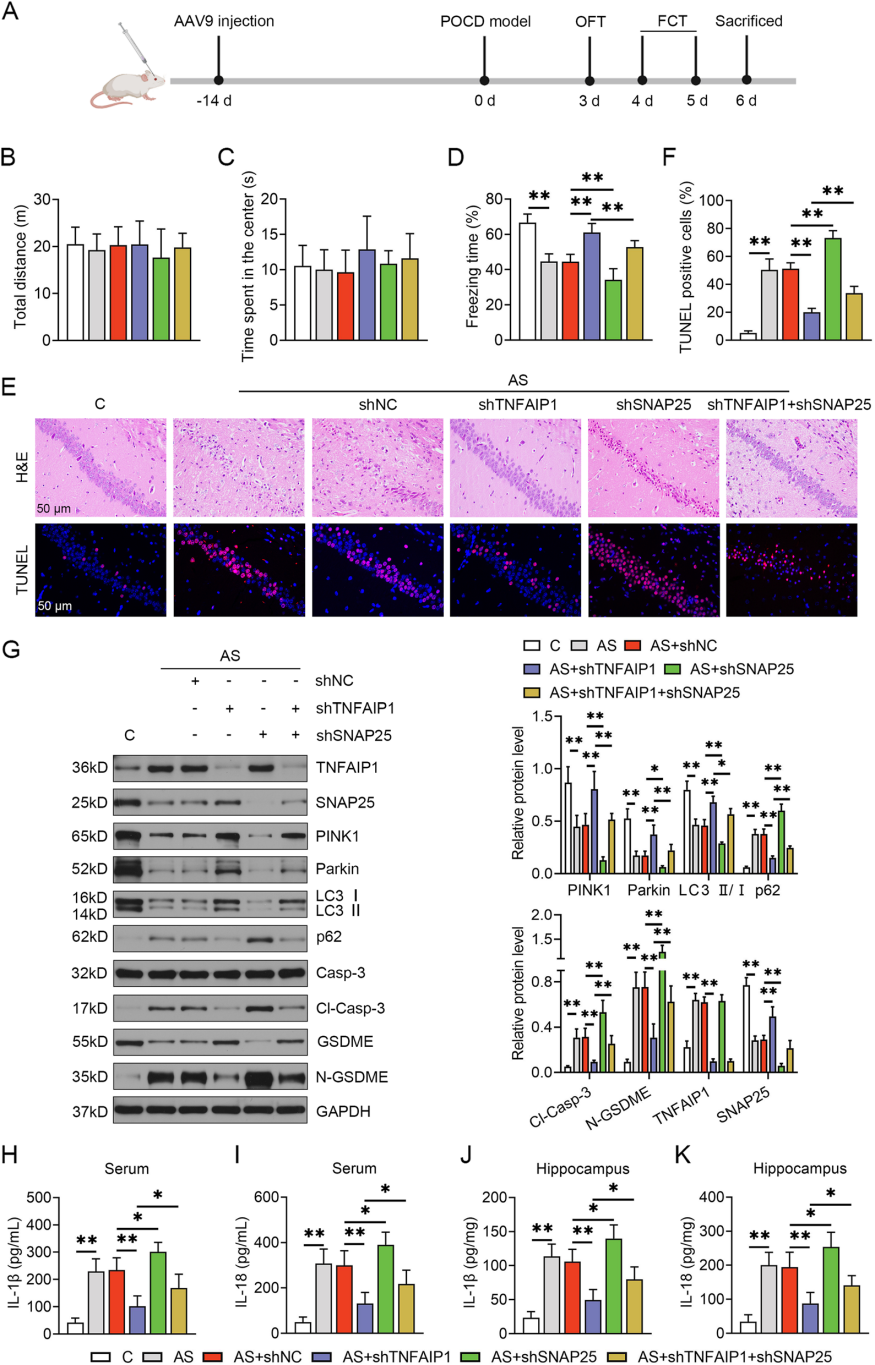
TNFAIP1 knockdown ameliorates postoperative cognitive impairment, which is abrogated by SNAP25 knockdown. **A** Experimental design. Two weeks after AAV9 injection, mice were subjected to laparotomy under isoflurane anesthesia. The OFT and FCT were performed from the third to fifth day. The hippocampus of different groups was harvested after behavioral assessment. The total moving distance (**B**) time spent in the center region (**C**) in the OFT. **D** Freezing response of mice was assessed by contextual fear conditioning as the proportion of immobility (*n* = 12). ^**^P < 0.01, one-way ANOVA test. **E** H&E (400 × , bar: 50 μm) and TUNEL staining (400 × , bar: 50 μm) of hippocampal CA1 samples following the indicated treatments. TUNEL-positive cells are shown in red, and nuclei are shown in blue. **F** The Percentage of TUNEL-positive cells in the hippocampi of mice (*n* = 3). ^**^P < 0.01, one-way ANOVA test. **G** Gel images of mitophagy- and pyroptosis-related proteins in the hippocampi microinjected with the indicated viruses. GAPDH served as a loading control (left panel). Quantification results normalized to GAPDH were represented as mean ± SEM (right panel, *n* = 4). ^*^P < 0.05, ^**^P < 0.01, one-way ANOVA test. The concentration of IL-1β and IL-18 was measured by ELISA in the serum (**H-I**) and hippocampi (**J-K**) of mice following the indicated treatments (*n* = 4). ^*^P < 0.05, ^**^P < 0.01, one-way ANOVA test

To elucidate the role of TNFAIP1 in the pathogenesis of POCD and whether SNAP25 blunts TNFAIP1-mediated phenotypes, AAV9-hSyn-shTNFAIP1 or AAV9-hSyn-shSNAP25 were used to map the hippocampal neurons in mice subjected to isoflurane/laparotomy, as illustrated in Fig. S7A-B. Successful transduction was confirmed by abundant green fluorescence distributed throughout the hippocampi (Fig. S7C). A schematic of the experimental protocol was shown in Fig. 5A. No significant differences were observed among groups regarding the total distance and duration of the center region in the open field test (Fig. 5B, C), indicating that isoflurane and laparotomy, regardless of neuron-specific knockdown of TNFAIP1 or SNAP25 pretreatment, did not alter spontaneous locomotor activity. Mice undergoing anesthesia and surgery showed a shorter duration of contextual freezing than the control group, suggesting that the POCD model was successfully established. TNFAIP1-KD mice showed more freezing behavior than the negative control group, while SNAP25-KD mice exhibited decreased freezing time. Simultaneous treatment with AAV9-hSyn-shTNFAIP1 and AAV9-hSyn-shSNAP25 resulted in worse hippocampus-dependent memory performance, as manifested by a lower percentage of freezing time compared to shTNFAIP1 alone (Fig. 5D). Hippocampal neurons in surgery-treated mice displayed irregular morphologies, such as crimson cytoplasm and denatured and necrotic nuclei. The AAV9-hSyn-shSNAP25 group exhibited more severe neuronal lesions, including nuclear pyknosis of ovoid shape and vague nuclear membrane, compared to the hippocampal neurons of surgery-treated mice. Nevertheless, selective neuronal depletion of TNFAIP1 restored the original morphology with intact, aligned neurons, which was counteracted by SNAP25 silencing (Fig. 5E-H&E). TUNEL staining revealed numerous TUNEL-positive cells in the hippocampi of mice undergoing laparotomy relative to that in the control group. Selective neuronal ablation of TNFAIP1 rescued surgery-induced cell death, whereas AAV9-hSyn-shSNAP25 further exacerbated this phenomenon. Concurrent neuron-specific inhibition of TNFAIP1 and SNAP25 significantly increased TUNEL-positive cells compared to AAV9-hSyn-shTNFAIP1 alone (Fig. 5E-TUNEL and F). Immunoblot assays showed that isoflurane and laparotomy remarkably diminished the hippocampal protein abundance of SNAP25, PINK1, Parkin, and LC3-II, but facilitated p62 accumulation and the cleavage of caspase-3 and GSDME. Upregulation of SNAP25, PINK1, Parkin, and LC3-II, in parallel with downregulation of p62, cleaved caspase-3, and N-GSDME occurred in the hippocampi of TNFAIP1-deficient mice following anesthesia and surgery. In addition to exacerbating surgery-triggered insufficient mitophagy and pyroptosis activation, AAV9-hSyn-shSNAP25 reversed the favorable effects of TNFAIP1 silencing on PINK1/Parkin signaling and inactivation of the caspase-3/GSDME axis (Fig. 5G). Unlike conventional protein secretion, the pro-inflammatory cytokines IL-1β and IL-18 are readily released through the large plasma membrane pores upon GSDME activation [34]. The concentration of IL-1β and IL-18 was prominently elevated in the serum and hippocampus isolated from POCD mice. Genetic suppression of TNFAIP1 significantly reduced the release of IL-1β and IL-18. In contrast, neuronal knockdown of SNAP25 further accelerated the release of these two cytokines. The augmented contents of IL-1β and IL-18 were found in the serum and hippocampus of TNFAIP1/SNAP25 double knockdown mice compared to those in the AAV9-hSyn-shTNFAIP1 alone (Fig. 5H-K). Taken together, these findings support the idea that TNFAIP1 silencing mitigates postoperative cognitive impairment, intensifies PINK1/Parkin-mediated mitophagy and restrains caspase-3/GSDME-mediated pyroptosis by stimulating SNAP25 protein expression.

## Discussion

In this study, we identified TNFAIP1 as a ubiquitin ligase responsible for the degradation of SNAP25. Through our investigations, we determined that the N-terminal portion of TNFAIP1, containing the BTB domain, interacted with SNAP25 and targeted it for K48-linked ubiquitination and subsequent degradation at Lys69. Our results showed that TNFAIP1 was substantially upregulated in the hippocampus of mice undergoing laparotomy with isoflurane anesthesia. However, neuron-specific knockdown of TNFAIP1 ameliorated surgery-elicited memory deficits, insufficient mitophagy dependent on PINK1/Parkin, and pyroptosis downstream of caspase-3/GSDME activation by stabilizing SNAP25.

Protein ubiquitination, a common posttranslational modification, plays a crucial role in neurodegeneration and synaptic plasticity related to learning [35, 36]. An antibody-based ubiquitome analysis has identified numerous ubiquitination events on synaptic proteins (e.g. SNAP25 and PSD95) in the rodent brain under physiological conditions [37]. It has been reported that the absence of CSPα knockout rendered SNAP25 susceptible to degradation in a ubiquitin–proteasome manner, and impaired the incorporation of SNAP25 into SNARE complexes [38]. Another E3 ligase, TRIM9, has been documented to interact with SNAP25 and hinder SNARE complex formation independently of the proteasome [39]. However, limited studies have investigated ubiquitin ligases responsible for SNAP25 degradation. By querying in the UbiBrowser database and considering criteria based on single-cell RNA sequencing data (GSE67835) and their association with inflammation [27], TNFAIP1 was predicted as a potential regulator for SNAP25. The interaction between TNFAIP1 and SNAP25 was confirmed by Co-IP as well as GST pull-down assays. BTB domain-containing proteins act as important determinants of substrate recognition to drive assembly of E3 ligase complexes involving the scaffold protein Cullin3 (CUL3) and the RING finger protein RING-box protein 1 (RBX1) [40]. Although the vast majority of evidence suggests that the N-terminal BTB domain binds to CUL3 and the C-terminal domain binds to substrates [40–42], several studies support the involvement of the BTB domain in substrate interaction [43, 44]. The C-terminal moiety (97–316 aa) of TNFAIP1 was required for its interaction with CSNK2B [14], but our research highlighted that the N-terminal segment (1–96 aa) encompassing the BTB domain had a strong affinity for SNAP25 binding. In line with previous investigations reporting ubiquitin-dependent substrate degradation mediated by TNFAIP1 [14, 15], we also detected its capacity to orchestrate K48-linked polyubiquitylation of SNAP25. The amino acid sequence of SNAP25 was identical among Homo sapiens, Mus musculus, and Rattus norvegicus according to the multiple sequence alignment tool "Align" provided by UniProt [45]. A rat brain ubiquitome analysis has identified K40, K69, K72, K76, and K103 as potential ubiquitination sites without experimental verification [37]. Our results highlighted the specific interaction between TNFAIP1 and K69-ubiquitylated SNAP25.

TNFAIP1 overexpression has been shown to contribute to β-amyloid (Aβ)-induced weak cell viability, ROS accumulation and cell death in SH-SY5Y cells, while its silencing has the opposite effect [17]. TNFAIP1 depletion has been found to enhance cell viability and protect N2a cells from apoptosis caused by plasticizer or formaldehyde stress [18, 19]. Furthermore, TNFAIP1 knockdown in PC12 cells can enhance cell viability, and it also possesses antioxidant and ferroptosis inhibition properties [46]. In our study, TNFAIP1-deficient SH-SY5Y cells exhibited a small proportion of Annexin V/PI double-positive cells, increased cell viability, and low ROS content, while TNFAIP1-overexpressing HT22 cells showed reduced cell viability and excessive oxidative stress and pyroptosis. Disruption of mitochondrial membrane potential (MMP, ∆Ψm) is a hallmark of mitophagy [47]. TNFAIP1 has been suggested to affect ∆Ψm and autophagy in SH-SY5Y cells treated with Aβ [17, 48], indicating its presumable involvement in neuronal mitophagy. Our in vitro and in vivo data ascertained that TNFAIP1 silencing enhanced PINK1/Parkin-dependent mitophagy, while TNFAIP1 overexpression dampened the expression of PINK1, Parkin, LC3-II, leading to the accumulation of p62. Consistent with the favorable effects of TNFAIP1 on caspase 1/3/7 activity in macrophages or colorectal cancer cells [49, 50], we found that TNFAIP1 promoted caspase-3 cleavage and subsequent generation of the N-terminal fragment of GSDME both in vitro and in vivo. Through rescue experiments in HT22 cells and mice, we corroborated that TNFAIP1 governed PINK1/Parkin-mediated mitophagy and caspase-3/GSDME-mediated pyroptosis by negatively regulating SNAP25 protein abundance.

Albeit little literature on the role of TNFAIP1 in the modulation of cognitive function, it has been implicated in reducing dendritic spine density, which contributes to long-term cognitive abnormalities [51, 52]. Since SNAP25 ameliorates POCD by boosting PINK1-dependent mitophagy and dampening pyroptosis [5], we explored the impact of TNFAIP1 on learning and memory. The OFT results emphasized minimal impact of AAV9-hSyn-shTNFAIP1 or AAV9-hSyn-shSNAP25 on the locomotor activity of mice. However, neuron-specific knockdown of TNFAIP1 significantly improved memory performance in the contextual fear conditioning test, which was counteracted by SNAP25 depletion. Recent evidence has shown that shRNA against TNFAIP1 reduces TUNEL positive cells in the myocardium subjected to ischemia/reperfusion [53], which aligned with our results. Pyroptosis is a programmed form of cell death culminating in IL-1β and IL-18 secretion [54]. TNFAIP1 overexpression can stimulate the release of IL-1β, IL-6, and TNF-α from LPS-treated BV-2 microglia and oxidized low-density lipoprotein-primed THP-1 macrophages [55, 56]. Conversely, TNFAIP1 silencing blocks the detrimental effects of these pro-inflammatory cytokines on PC12 cells and myocardium subjected to ischemia/reperfusion injury [46, 53]. In addition to reducing IL-1β release, AAV9-hSyn-shTNFAIP1 therapy markedly diminished IL-18 concentration in the serum and hippocampus following anesthesia and surgery. Additionally, neuron-specific knockdown of SNAP25 abolished this beneficial effect.

Our study has limitations. There has been no exact evidence that aseptic surgical condition can cause how high serum LPS levels. LPS at the concentration of 1 μg/mL in neurons may not entirely chime with animal models. Better design on in vitro models needs to be taken into account in the future. Most animal studies do not precisely imitate clinical scenario of POCD. Not all surgical patients will suffer from POCD. However, experimental design of animal studies follows the principle of uniformity. Owing to the concern of learning influence if rodents receive the repeated paradigms of learning and memory, researchers do not generally introduce preoperative and postoperative cognitive tests to identify individual animals that develop memory impairment after surgery [57]. Zhong et al. proposed a novel individual-based diagnosis of POCD based on cognitive fluctuations from pre-surgery and post-surgery [58], which may be a promising alternative for performing in vivo assays. Since we employed male animals in our study to reduce potential impact of estrogen and progesterone on cognitive ability in females, the effects of TNFAIP1 on surgery-induced cognitive impairment in female mice are not known.

## Conclusions

In conclusion, our study provided insights into the ubiquitination mechanism that regulated the SNARE protein SNAP25, which may be critical for PINK1/Parkin-dependent mitophagy as well as caspase-3/GSDME-dependent pyroptosis. The results of this study could improve our understanding of the pathophysiology of POCD and offer a prospective therapeutic target.

### Supplementary Information

**Additional file 1: Table S1.** The putative E3 ubiquitin ligase predicted by UbiBrowser. **Fig S1.** (A) The amino acid sequence of SNAP25. Pale blue denotes lysines (K). Pink denotes ubiquitination sites of SNAP25 predicted by CKSAAP_UbSite webserver: K69, K184, K189 and K201. (B) Prediction score of each lysine. **Fig S2.** (A) SH-SY5Y cells were treated with siRNA negative control or three specific siRNAs against TNFAIP1 for 36 h. The most efficient fragment was screened using RT-qPCR (*n* = 3). **P < 0.01 vs siNC. GAPDH served as a housekeeping gene. Three potential siRNA sequences targeting human TNFAIP1 mRNA (NM_021137): siTNFAIP1#1 (5′-GGUUGGGCAACAAGUAUGUTT-3′), siTNFAIP1#2 (5′-CAAAGUAUUACCUCAUCCATT-3′), siTNFAIP1#3 (5′-CCGAAUCUAUGAGGAGACATT-3′). The negative control siRNA sequence was 5′-GAGTATGAGTCGGATGACGTAGCCA-3′. (B) CCK-8 assay showing the effect of siTNFAIP1 on SH-SY5Y cell viability (*n* = 3). *P < 0.05, **P < 0.01, one-way ANOVA test. **Fig S3.** Densitometric quantification of WB in Fig 3D (*n* = 4). *P < 0.05, **P < 0.01, one-way ANOVA test. **Fig S4.** (A) WB analysis confirmed that HT22 cells successfully overexpressed TNFAIP1 (*n* = 3). **P < 0.01, one-way ANOVA test. (B) CCK-8 assay showing the effect of TNFAIP1 on HT22 cell viability (*n* = 3). *P < 0.05, **P < 0.01, one-way ANOVA test. **Fig S5.** Densitometric quantification of WB in Fig 3H (*n* = 4). *P < 0.05, **P < 0.01, one-way ANOVA test. **Fig S6.** (A) WB analysis confirmed that HT22 cells successfully overexpressed SNAP25 (*n* = 3). **P < 0.01, one-way ANOVA test. (B) CCK-8 assay showing the effect of TNFAIP1 and SNAP25 on HT22 cell viability (*n* = 3). **P < 0.01, one-way ANOVA test. **Fig S7.** (A-B) HT22 cells were treated with shRNA negative control or three specific shRNAs against TNFAIP1 and SNAP25 for 36 h. The most efficient fragment was screened using WB (*n* = 3). **P < 0.01 vs shNC. GAPDH served as a loading control. Three potential shRNA sequences targeting mouse TNFAIP1 (NM_009395) and SNAP25 (NM_011428): shTNFAIP1#1 (5′-GGAAAGCACTTTGGCACCATCTTGA-3′), shTNFAIP1#2 (5′-CAGGCTCATTGAATCCTCCACAAAG-3′), shTNFAIP1#3 (5′-CGTCGCATTCATGTCAAGCGCTATA-3′); shSNAP25#1 (5′-GAGAGTAAAGATGCTGGCATCAGGA-3′), shSNAP25#2 (5′-GAGGAAGGGATGGACCAAATCAATA-3′), shSNAP25#3 (5′-CAGAATCGCCAGATCGACAGGATCA-3′). The negative control shRNA sequence was 5′-GGATGGATCTACTTCACCAAGCGA-3′. (C) Representative immunofluorescence image of AAV9-hSyn-shTNFAIP1 and AAV9-hSyn-shSNAP25 infection in the mouse hippocampus (40×, bar: 200 μm).

## Data Availability

Data generated during the current study are available from the corresponding author upon reasonable request.
