# Supplementary material for: Neuronal-specific TNFAIP1 ablation attenuates postoperative cognitive dysfunction via targeting SNAP25 for K48-linked ubiquitination
Source: Cell Commun Signal. 2023 Dec 15;21:356. doi: 10.1186/s12964-023-01390-z (PMC10722859; doi:10.1186/s12964-023-01390-z)
Supplement: Supplementary file 2 — Additional file 1: Table S1. The putative E3 ubiquitin ligase predicted by UbiBrowser. Fig S1. (A) The amino acid sequence of SNAP25. Pale blue denotes lysines (K). Pink denotes ubiquitination sites of SNAP25 predicted by CKSAAP_UbSite webserver: K69, K184, K189 and K201. (B) Prediction score of each lysine. Fig S2. (A) SH-SY5Y cells were treated with siRNA negative control or three specific siRNAs against TNFAIP1 for 36 h. The most efficient fragment was screened using RT-qPCR (n = 3). **P < 0.01 vs siNC. GAPDH served as a housekeeping gene. Three potential siRNA sequences targeting human TNFAIP1 mRNA (NM_021137): siTNFAIP1#1 (5′-GGUUGGGCAACAAGUAUGUTT-3′), siTNFAIP1#2 (5′-CAAAGUAUUACCUCAUCCATT-3′), siTNFAIP1#3 (5′-CCGAAUCUAUGAGGAGACATT-3′). The negative control siRNA sequence was 5′-GAGTATGAGTCGGATGACGTAGCCA-3′. (B) CCK-8 assay showing the effect of siTNFAIP1 on SH-SY5Y cell viability (n = 3). *P < 0.05, **P < 0.01, one-way ANOVA test. Fig S3. Densitometric quantification of WB in Fig 3D (n = 4). *P < 0.05, **P < 0.01, one-way ANOVA test. Fig S4. (A) WB analysis confirmed that HT22 cells successfully overexpressed TNFAIP1 (n = 3). **P < 0.01, one-way ANOVA test. (B) CCK-8 assay showing the effect of TNFAIP1 on HT22 cell viability (n = 3). *P < 0.05, **P < 0.01, one-way ANOVA test. Fig S5. Densitometric quantification of WB in Fig 3H (n = 4). *P < 0.05, **P < 0.01, one-way ANOVA test. Fig S6. (A) WB analysis confirmed that HT22 cells successfully overexpressed SNAP25 (n = 3). **P < 0.01, one-way ANOVA test. (B) CCK-8 assay showing the effect of TNFAIP1 and SNAP25 on HT22 cell viability (n = 3). **P < 0.01, one-way ANOVA test. Fig S7. (A-B) HT22 cells were treated with shRNA negative control or three specific shRNAs against TNFAIP1 and SNAP25 for 36 h. The most efficient fragment was screened using WB (n = 3). **P < 0.01 vs shNC. GAPDH served as a loading control. Three potential shRNA sequences targeting mouse TNFAIP1 (NM_009395) and SNAP25 (NM_011428): shTNFAIP1#1 [file 12964_2023_1390_MOESM1_ESM.docx]

**Supplementary information**

Neuronal-specific TNFAIP1 ablation attenuates postoperative cognitive dysfunction via targeting SNAP25 for K48-linked ubiquitination

Table S1. The putative E3 ubiquitin ligase predicted by UbiBrowser

| Rank | Gene symbol | Gene description | Score |
| --- | --- | --- | --- |
| 1 | NEDD4 | E3 ubiquitin-protein ligase NEDD4 | 0.788 |
| 2 | SOCS5 | Suppressor of cytokine signaling 5 | 0.719 |
| 3 | MDM2 | E3 ubiquitin-protein ligase Mdm2 | 0.715 |
| 4 | STUB1 | E3 ubiquitin-protein ligase CHIP | 0.704 |
| 5 | CBX8 | Chromobox protein homolog 8 | 0.695 |
| 6 | CBL | E3 ubiquitin-protein ligase CBL | 0.686 |
| 7 | PAFAH1B1 | Platelet-activating factor acetylhydrolase IB subunit alpha | 0.681 |
| 8 | SMURF1 | E3 ubiquitin-protein ligase SMURF1 | 0.679 |
| 9 | SYVN1 | E3 ubiquitin-protein ligase synoviolin | 0.677 |
| 10 | NEDD4L | E3 ubiquitin-protein ligase NEDD4-like | 0.677 |
| 11 | GNB1 | Guanine nucleotide-binding protein G(I)/G(S)/G(T) subunit beta-1 | 0.675 |
| 12 | RPH3AL | Rab effector Noc2 | 0.671 |
| 13 | SYTL4 | Synaptotagmin-like protein 4 | 0.667 |
| 14 | PCGF2 | Polycomb group RING finger protein 2 | 0.665 |
| 15 | CBX4 | E3 SUMO-protein ligase CBX4 | 0.665 |
| 16 | BAZ1B | Tyrosine-protein kinase BAZ1B | 0.665 |
| 17 | HSPA8 | Heat shock cognate 71 kDa protein | 0.653 |
| 18 | BARD1 | BRCA1-associated RING domain protein 1 | 0.649 |
| 19 | VHL | Von Hippel-Lindau disease tumor suppressor | 0.641 |
| 20 | IBTK | Inhibitor of Bruton tyrosine kinase | 0.640 |
| 21 | RING1 | E3 ubiquitin-protein ligase RING1 | 0.640 |
| 22 | MARCH9 | E3 ubiquitin-protein ligase MARCH9 | 0.633 |
| 23 | RNF216 | E3 ubiquitin-protein ligase RNF216 | 0.630 |
| 24 | MNAT1 | CDK-activating kinase assembly factor MAT1 | 0.628 |
| 25 | EP300 | Histone acetyltransferase p300 | 0.628 |
| 26 | CREBBP | CREB-binding protein | 0.628 |
| 27 | PML | Protein PML | 0.626 |
| 28 | TRIM27 | Zinc finger protein RFP | 0.622 |
| 29 | RANBP2 | E3 SUMO-protein ligase RanBP2 | 0.617 |
| 30 | TNFAIP1 | BTB/POZ domain-containing adapter for CUL3-mediated RhoA degradation protein 2 | 0.617 |
| 31 | CRYAB | Alpha-crystallin B chain | 0.617 |
| 32 | PPIL2 | Peptidyl-prolyl cis-trans isomerase-like 2 | 0.617 |
| 33 | CADPS2 | Calcium-dependent secretion activator 2 | 0.615 |
| 34 | WDTC1 | WD and tetratricopeptide repeats protein 1 | 0.615 |
| 35 | STC1 | Stanniocalcin-1 | 0.615 |
| 36 | SMURF2 | E3 ubiquitin-protein ligase SMURF2 | 0.611 |
| 37 | ASB2 | Ankyrin repeat and SOCS box protein 2 | 0.610 |
| 38 | PARK2 | E3 ubiquitin-protein ligase parkin | 0.609 |
| 39 | SOCS7 | Suppressor of cytokine signaling 7 | 0.609 |
| 40 | TEP1 | Telomerase protein component 1 | 0.605 |
| 41 | CISH | Cytokine-inducible SH2-containing protein | 0.605 |
| 42 | UBE3C | Ubiquitin-protein ligase E3C | 0.604 |
| 43 | FZR1 | Fizzy-related protein homolog | 0.604 |
| 44 | TTC3 | E3 ubiquitin-protein ligase TTC3 | 0.604 |


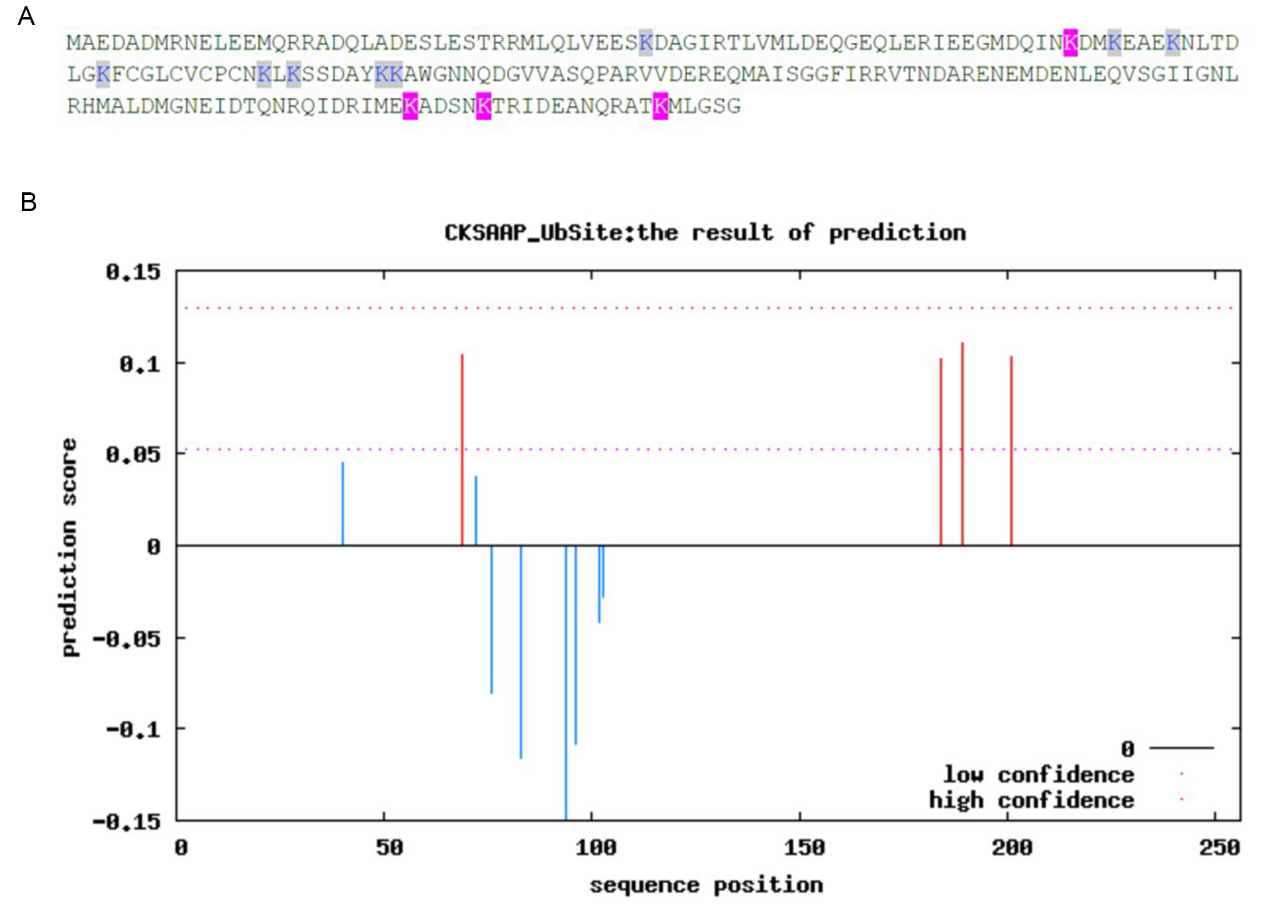


**Fig S1.** **(A)** The amino acid sequence of SNAP25. Pale blue denotes lysines (K). Pink denotes ubiquitination sites of SNAP25 predicted by CKSAAP_UbSite webserver: K69, K184, K189 and K201. **(B)** Prediction score of each lysine.


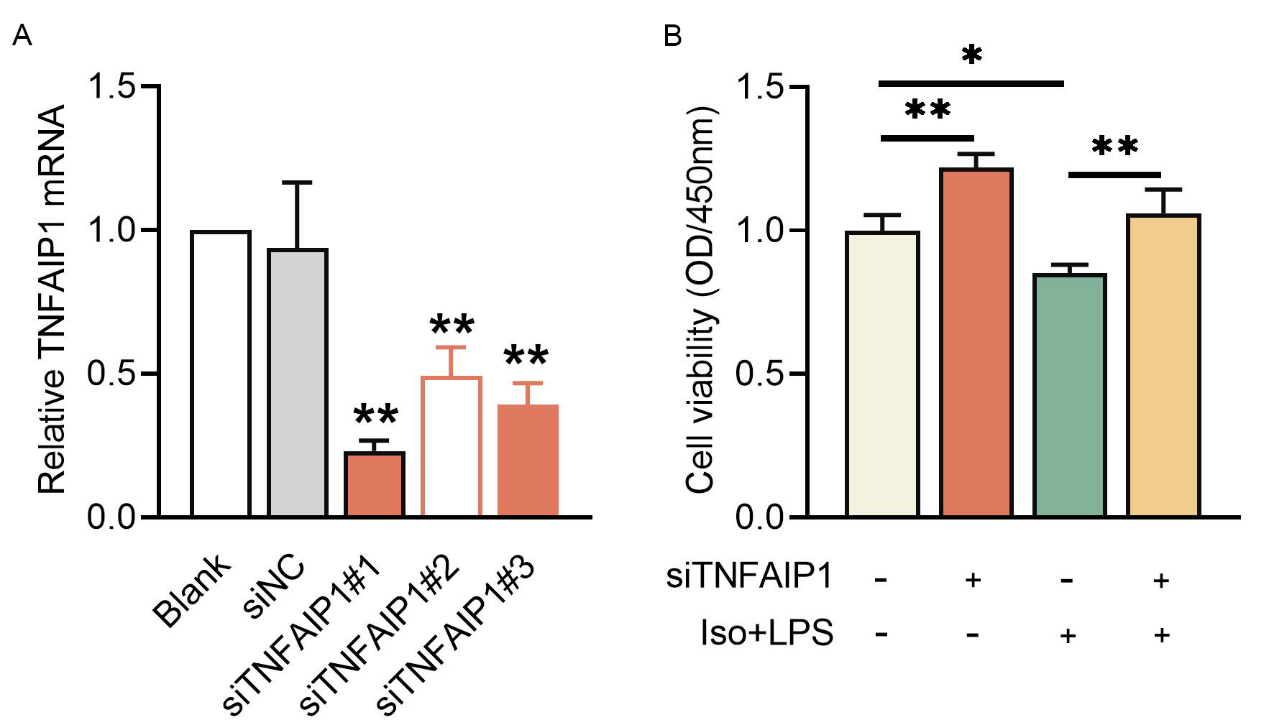


**Fig S2.** **(A)** SH-SY5Y cells were treated with siRNA negative control or three specific siRNAs against TNFAIP1 for 36 h. The most efficient fragment was screened using RT-qPCR (n = 3). ^**^*P* < 0.01 vs siNC. GAPDH served as a housekeeping gene. Three potential siRNA sequences targeting human TNFAIP1 mRNA (NM_021137): siTNFAIP1#1 (5′-GGUUGGGCAACAAGUAUGUTT-3′), siTNFAIP1#2 (5′-CAAAGUAUUACCUCAUCCATT-3′), siTNFAIP1#3 (5′-CCGAAUCUAUGAGGAGACATT-3′). The negative control siRNA sequence was 5′-GAGTATGAGTCGGATGACGTAGCCA-3′. **(B)** CCK-8 assay showing the effect of siTNFAIP1 on SH-SY5Y cell viability (n = 3). ^*^*P* < 0.05, ^**^*P* < 0.01, one-way ANOVA test.


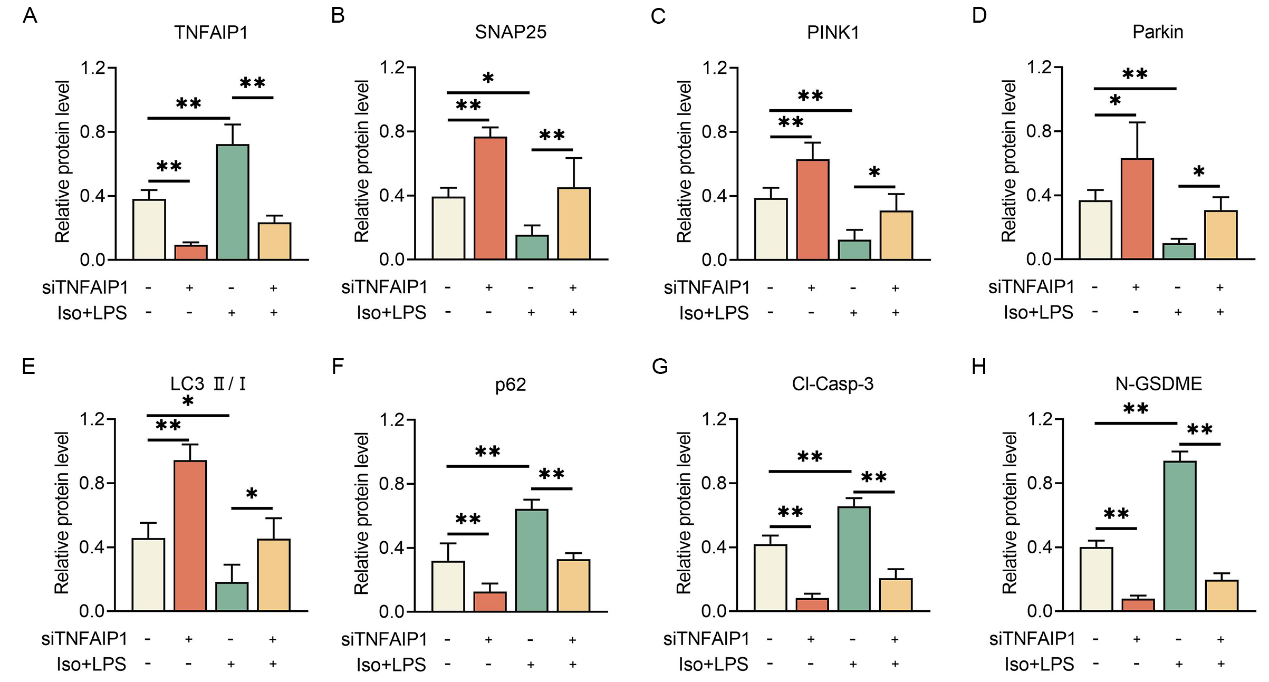


**Fig S3.** Densitometric quantification of WB in Fig 3D (n = 4). ^*^*P* < 0.05, ^**^*P* < 0.01, one-way ANOVA test.


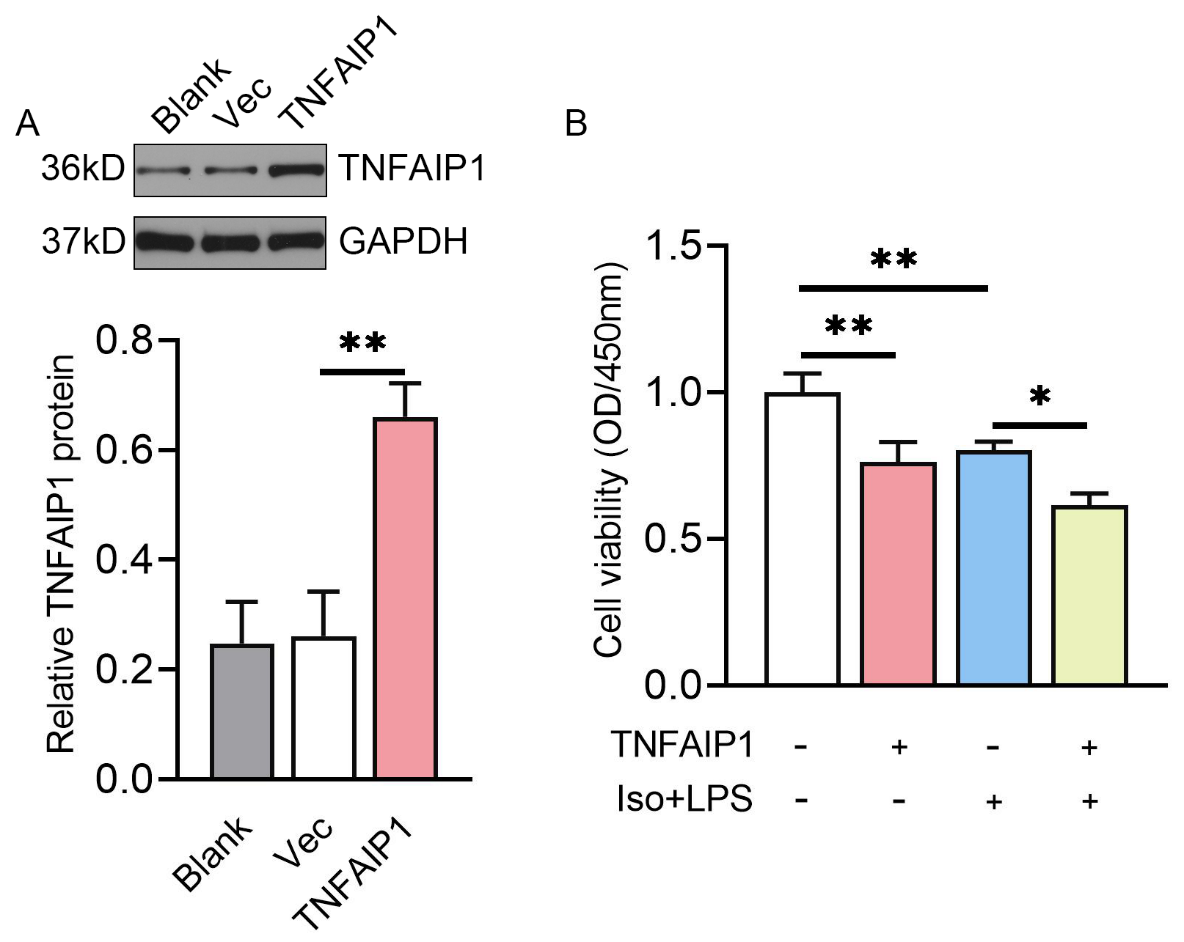


**Fig S4. (A)** WB analysis confirmed that HT22 cells successfully overexpressed TNFAIP1 (n = 3). ^**^*P* < 0.01, one-way ANOVA test. (B) CCK-8 assay showing the effect of TNFAIP1 on HT22 cell viability (n = 3). ^*^*P* < 0.05, ^**^*P* < 0.01, one-way ANOVA test.


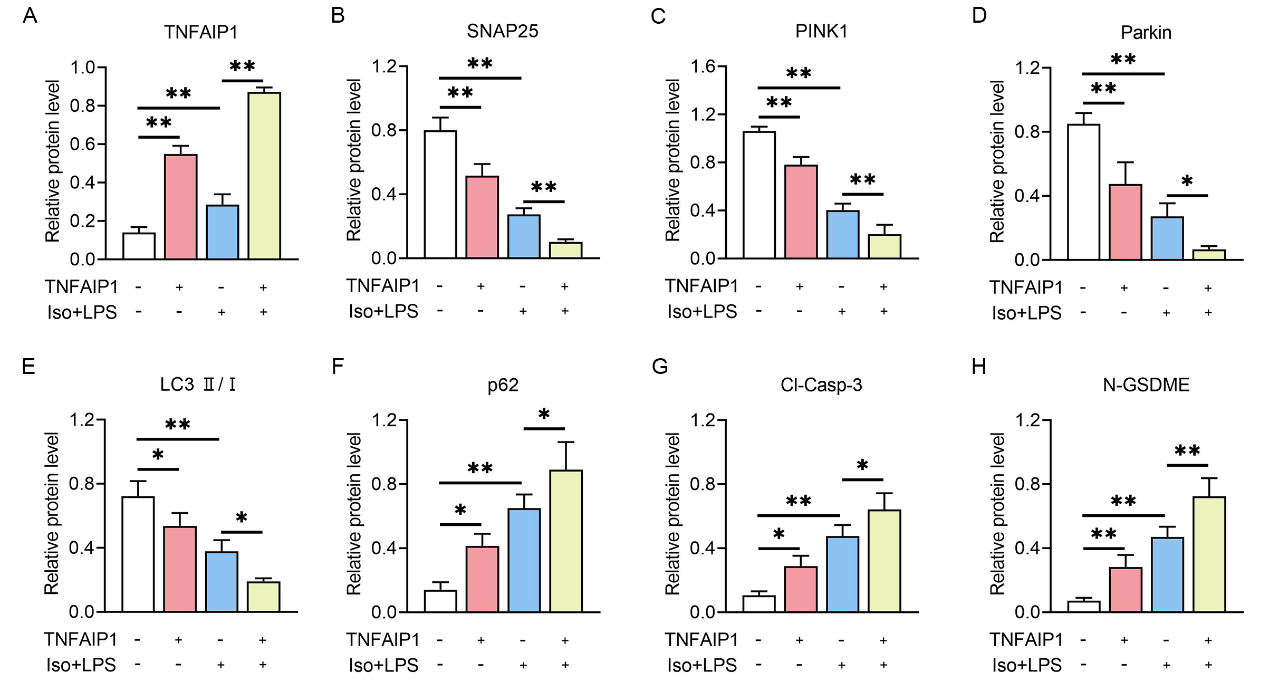


**Fig S5.** Densitometric quantification of WB in Fig 3H (n = 4). ^*^*P* < 0.05, ^**^*P* < 0.01, one-way ANOVA test.


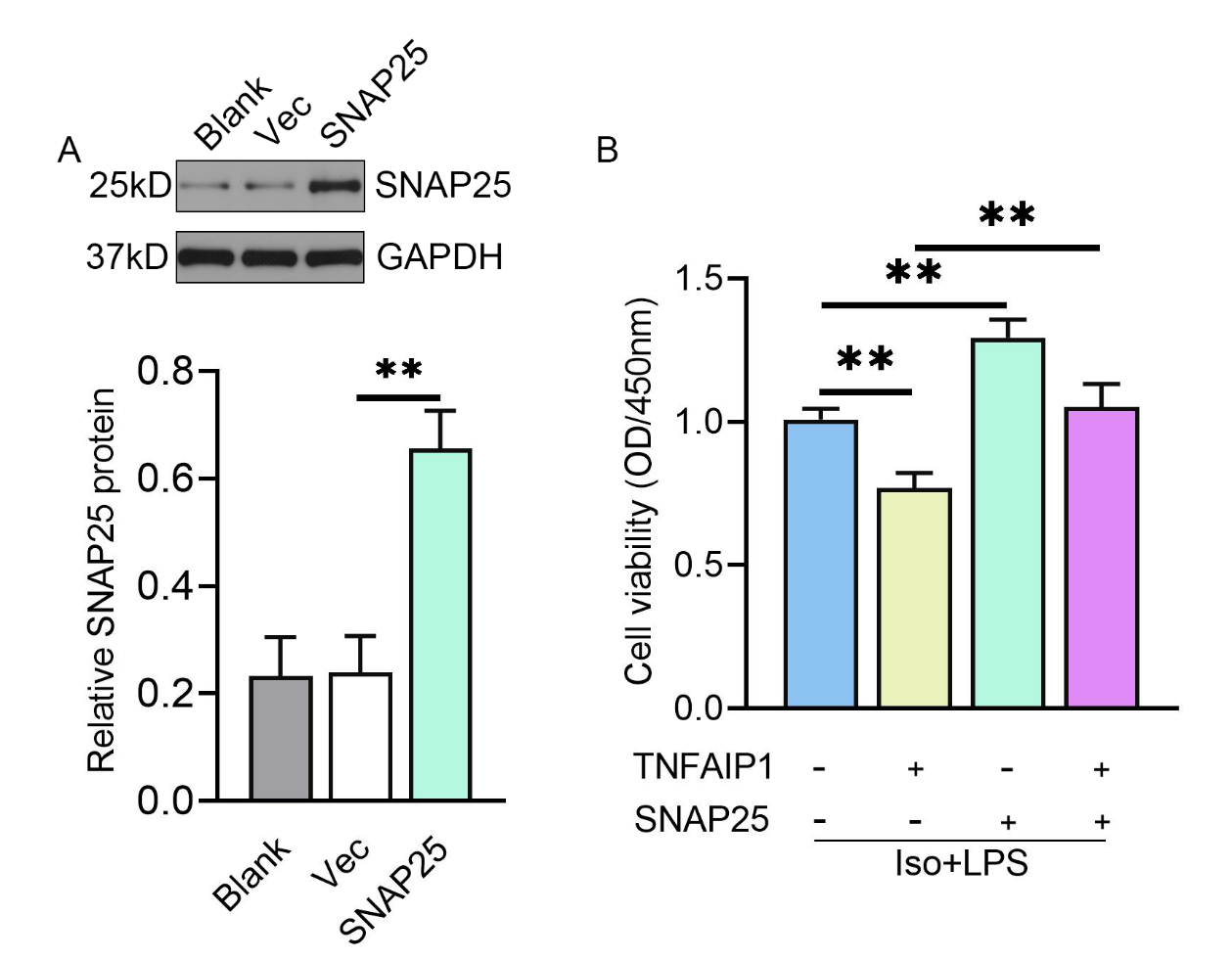


**Fig S6. (A)** WB analysis confirmed that HT22 cells successfully overexpressed SNAP25 (n = 3). ^**^*P* < 0.01, one-way ANOVA test. (B) CCK-8 assay showing the effect of TNFAIP1 and SNAP25 on HT22 cell viability (n = 3). ^**^*P* < 0.01, one-way ANOVA test.


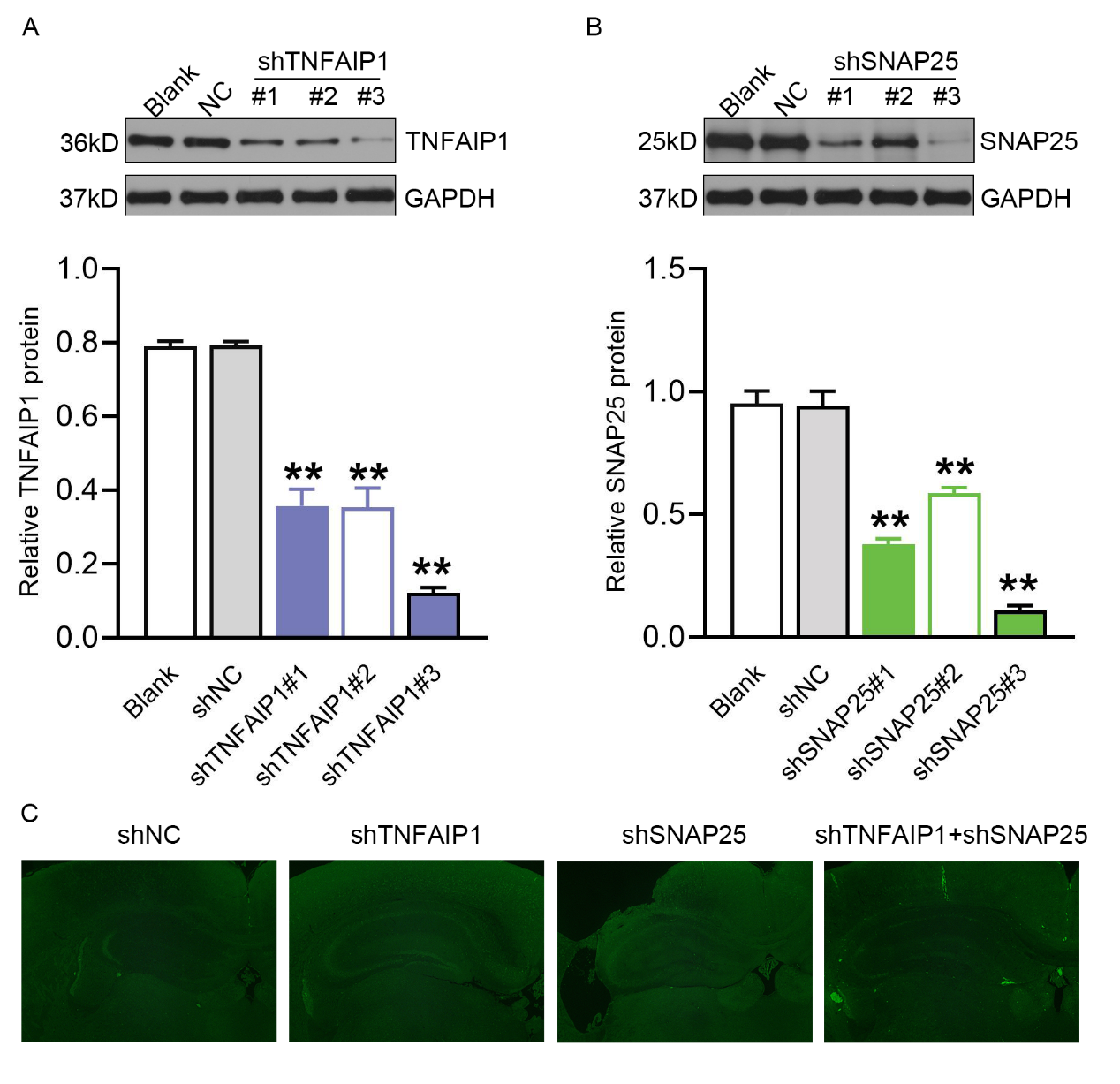


**Fig S7. (A-B)** HT22 cells were treated with shRNA negative control or three specific shRNAs against TNFAIP1 and SNAP25 for 36 h. The most efficient fragment was screened using WB (n = 3). ^**^*P* < 0.01 vs shNC. GAPDH served as a loading control. Three potential shRNA sequences targeting mouse TNFAIP1 (NM_009395) and SNAP25 (NM_011428): shTNFAIP1#1 (5′-GGAAAGCACTTTGGCACCATCTTGA-3′), shTNFAIP1#2 (5′-CAGGCTCATTGAATCCTCCACAAAG-3′), shTNFAIP1#3 (5′-CGTCGCATTCATGTCAAGCGCTATA-3′); shSNAP25#1 (5′-GAGAGTAAAGATGCTGGCATCAGGA-3′), shSNAP25#2 (5′-GAGGAAGGGATGGACCAAATCAATA-3′), shSNAP25#3 (5′-CAGAATCGCCAGATCGACAGGATCA-3′). The negative control shRNA sequence was 5′-GGATGGATCTACTTCACCAAGCGA-3′. (C) Representative immunofluorescence image of AAV9-hSyn-shTNFAIP1 and AAV9-hSyn-shSNAP25 infection in the mouse hippocampus (40×, bar: 200 μm).

**The full uncropped Gels and Blots image(s) as follow:**


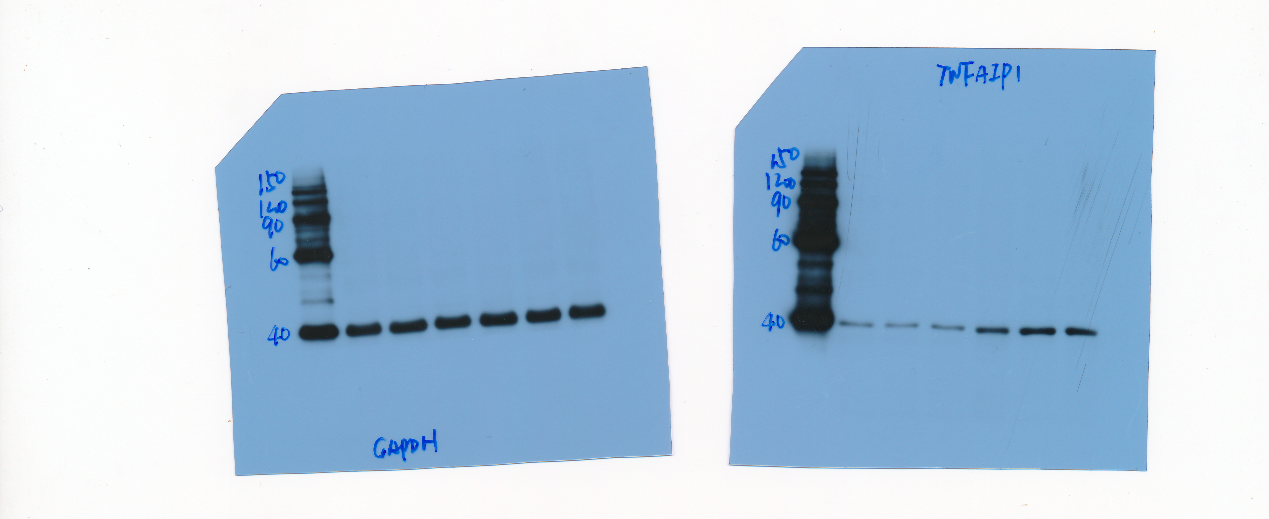


Fig 1B


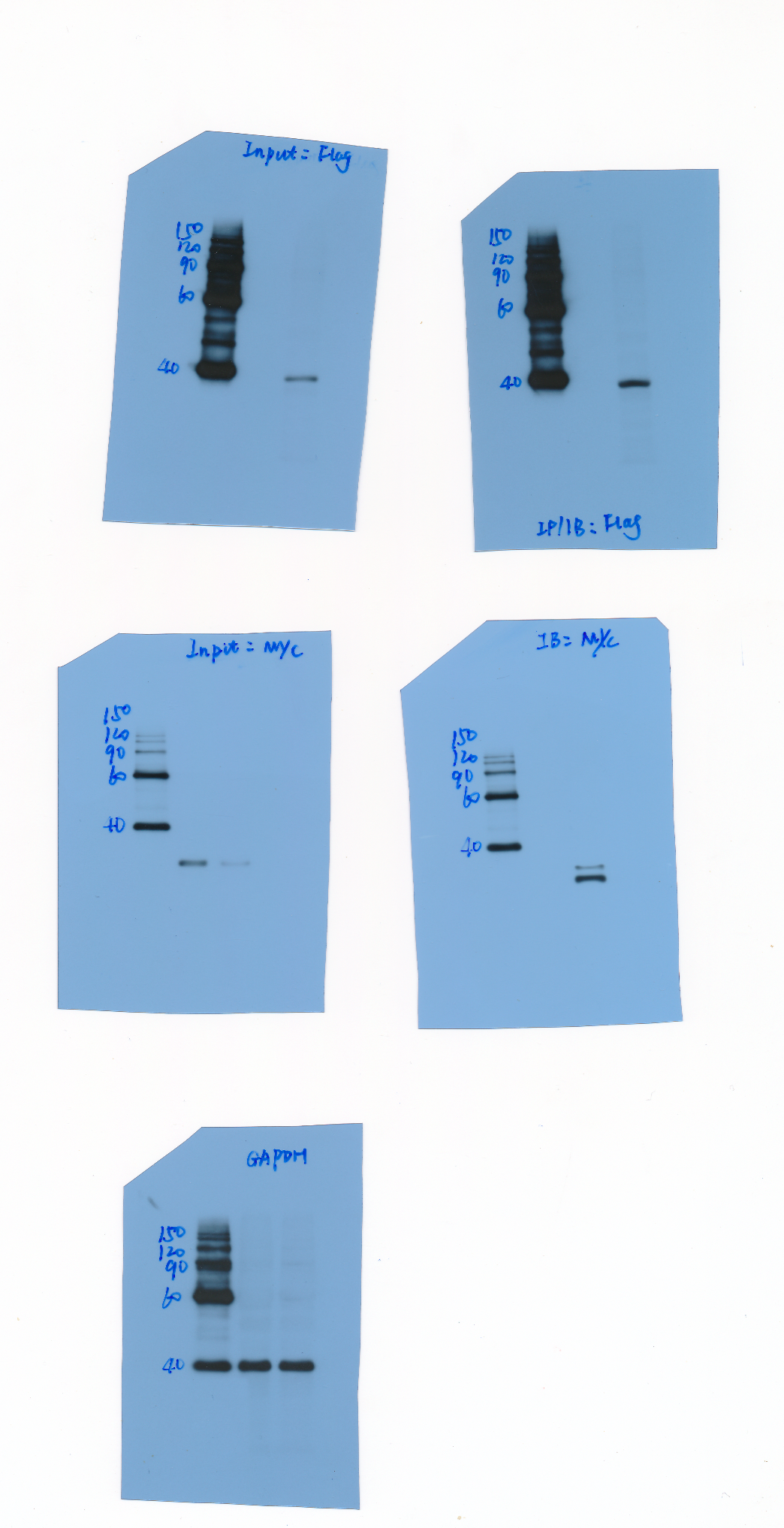


Fig 1C


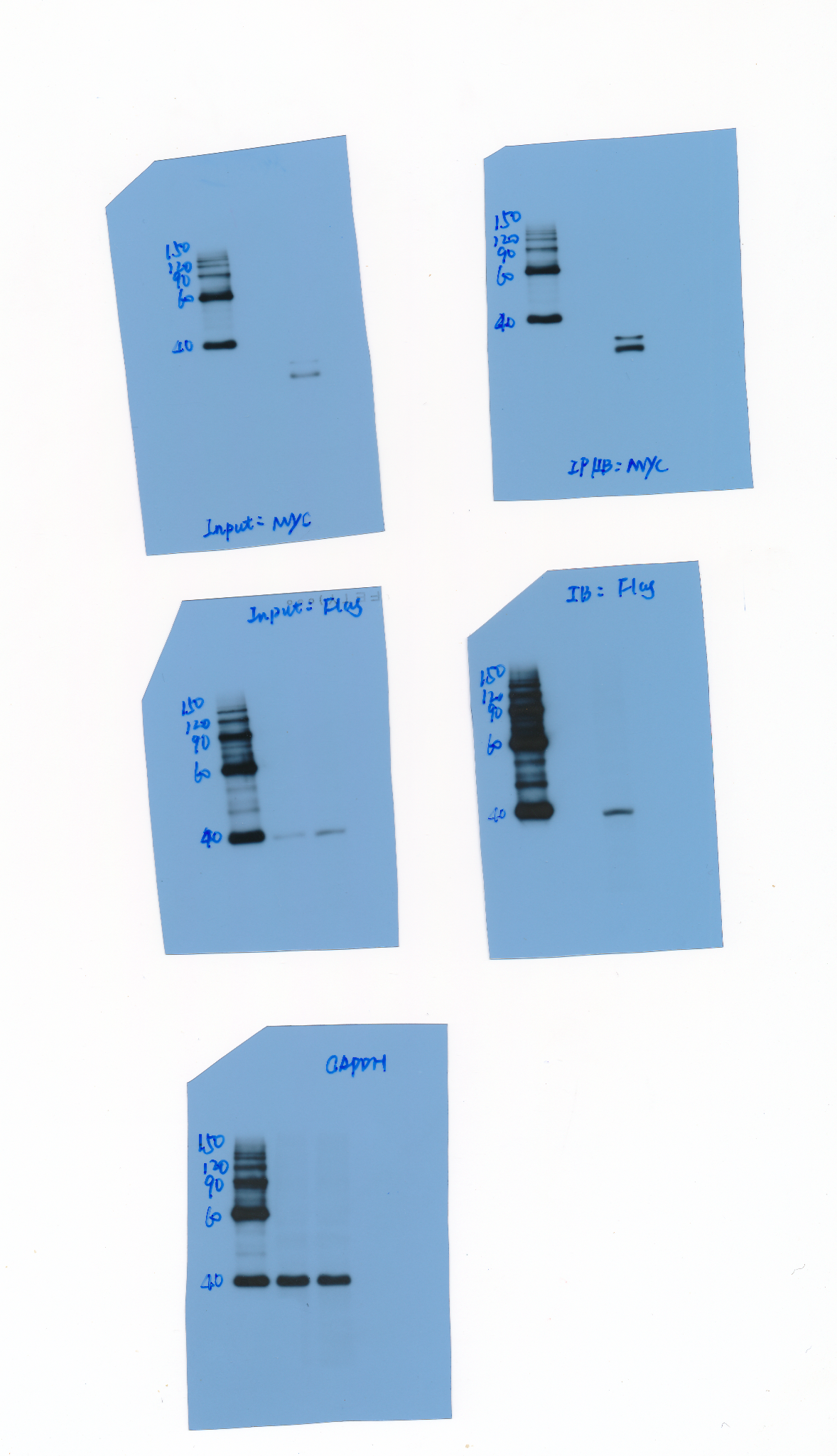


Fig 1D


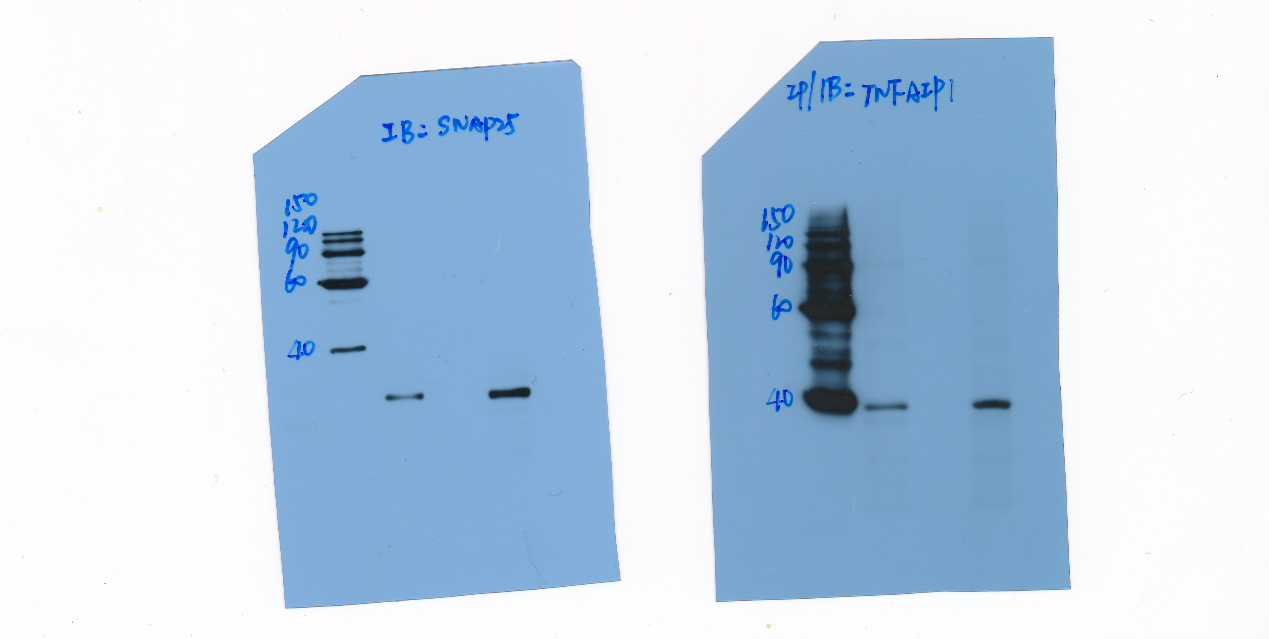


Fig 1E


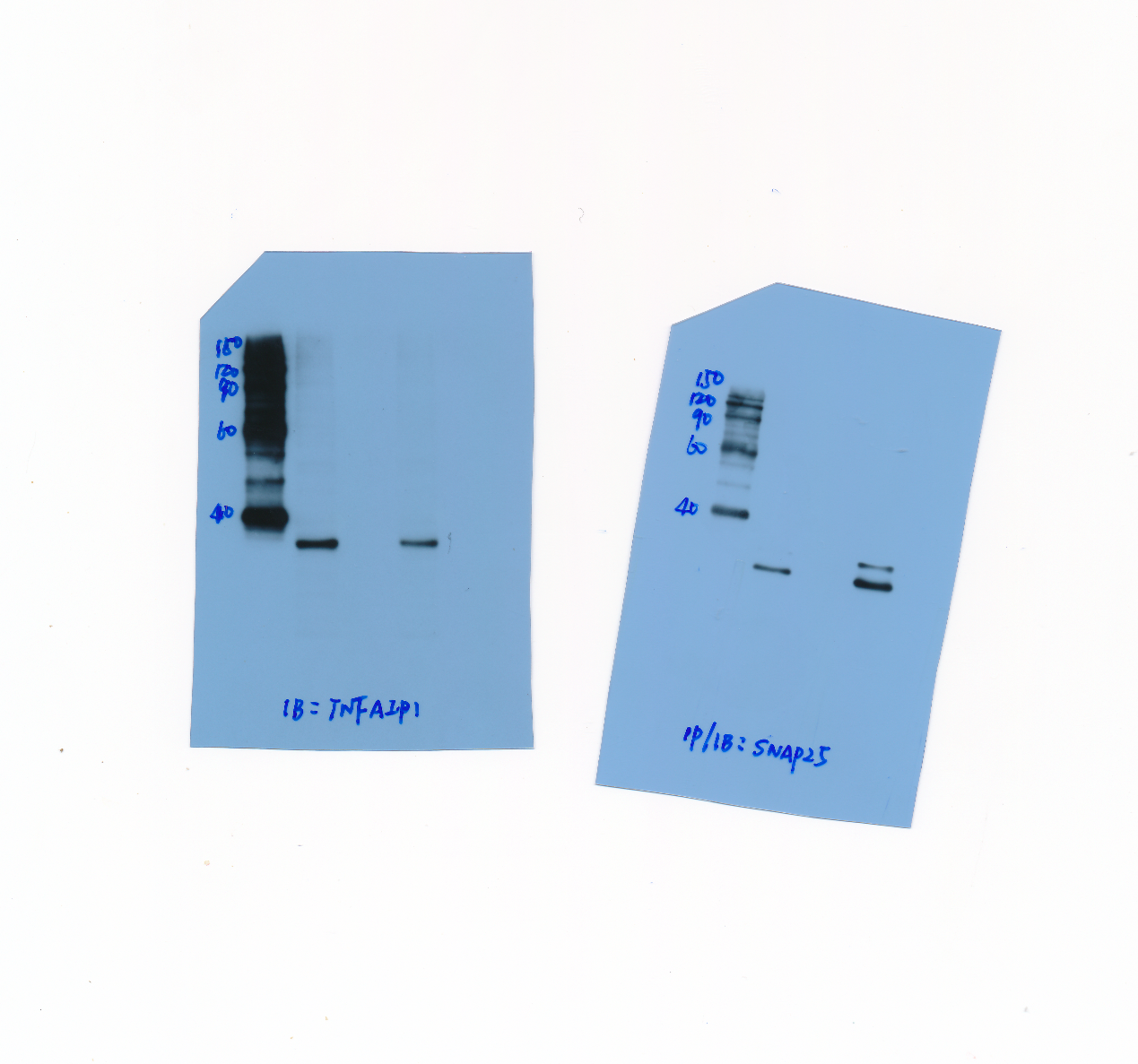


Fig 1F


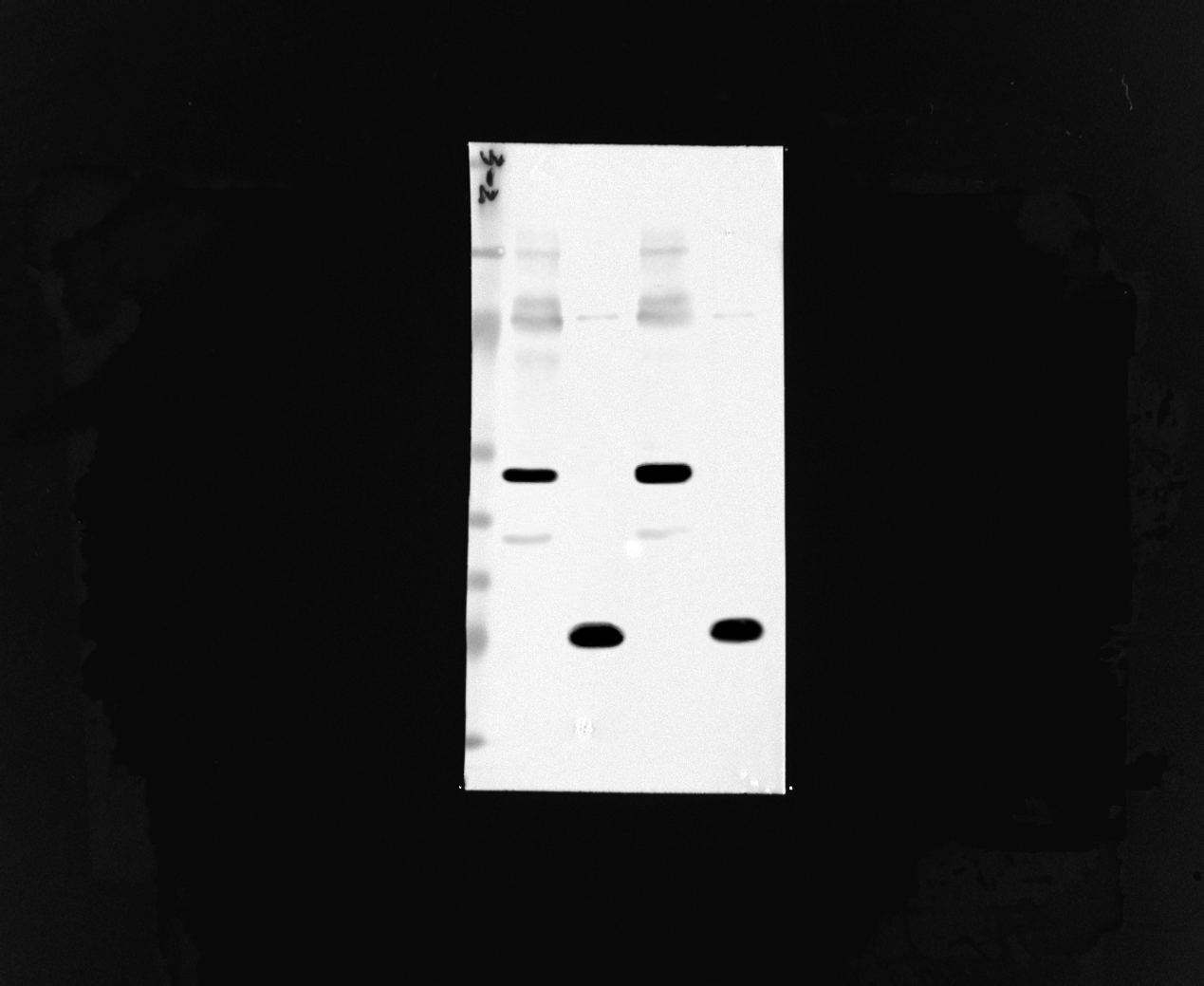

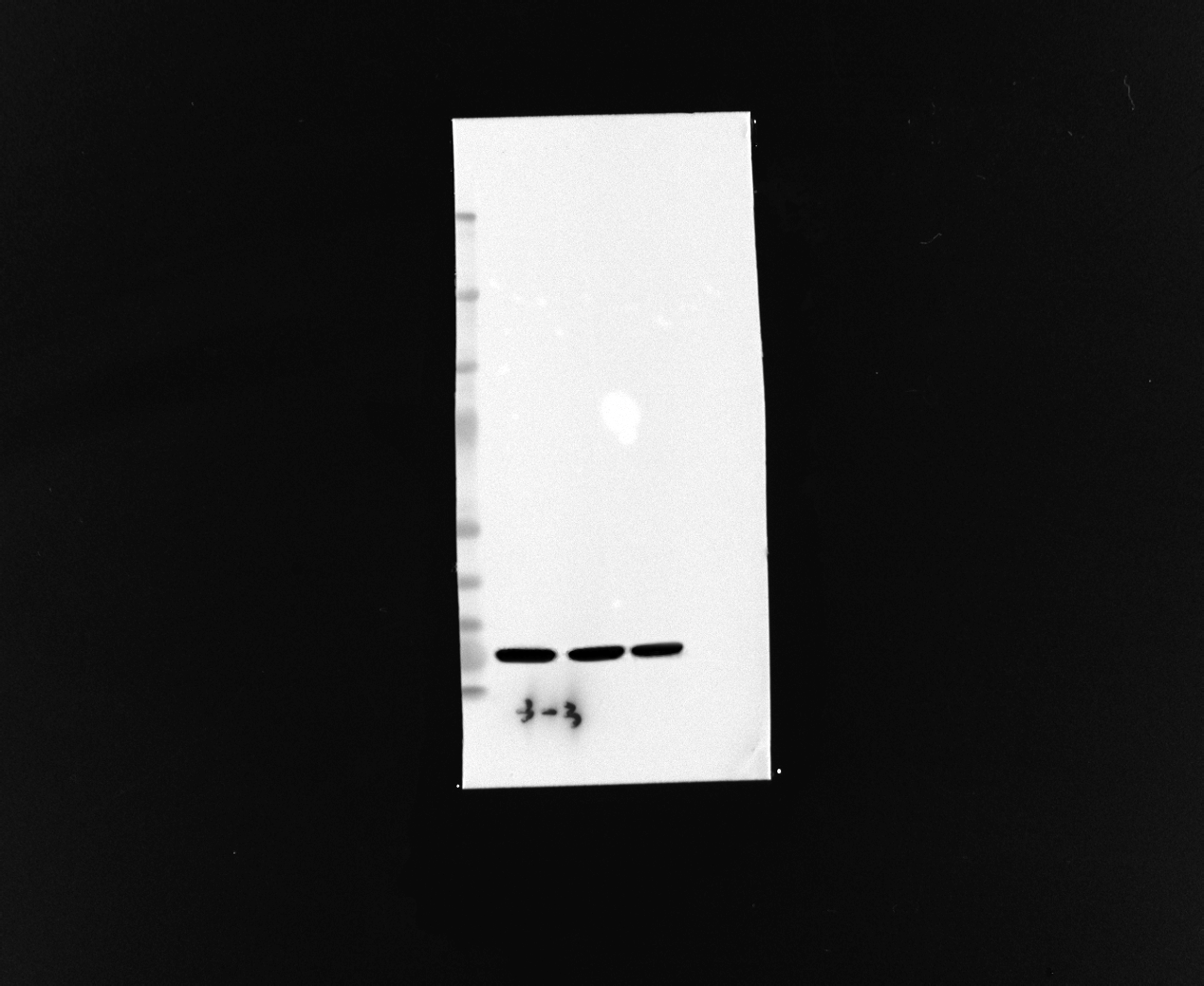


Fig 1G


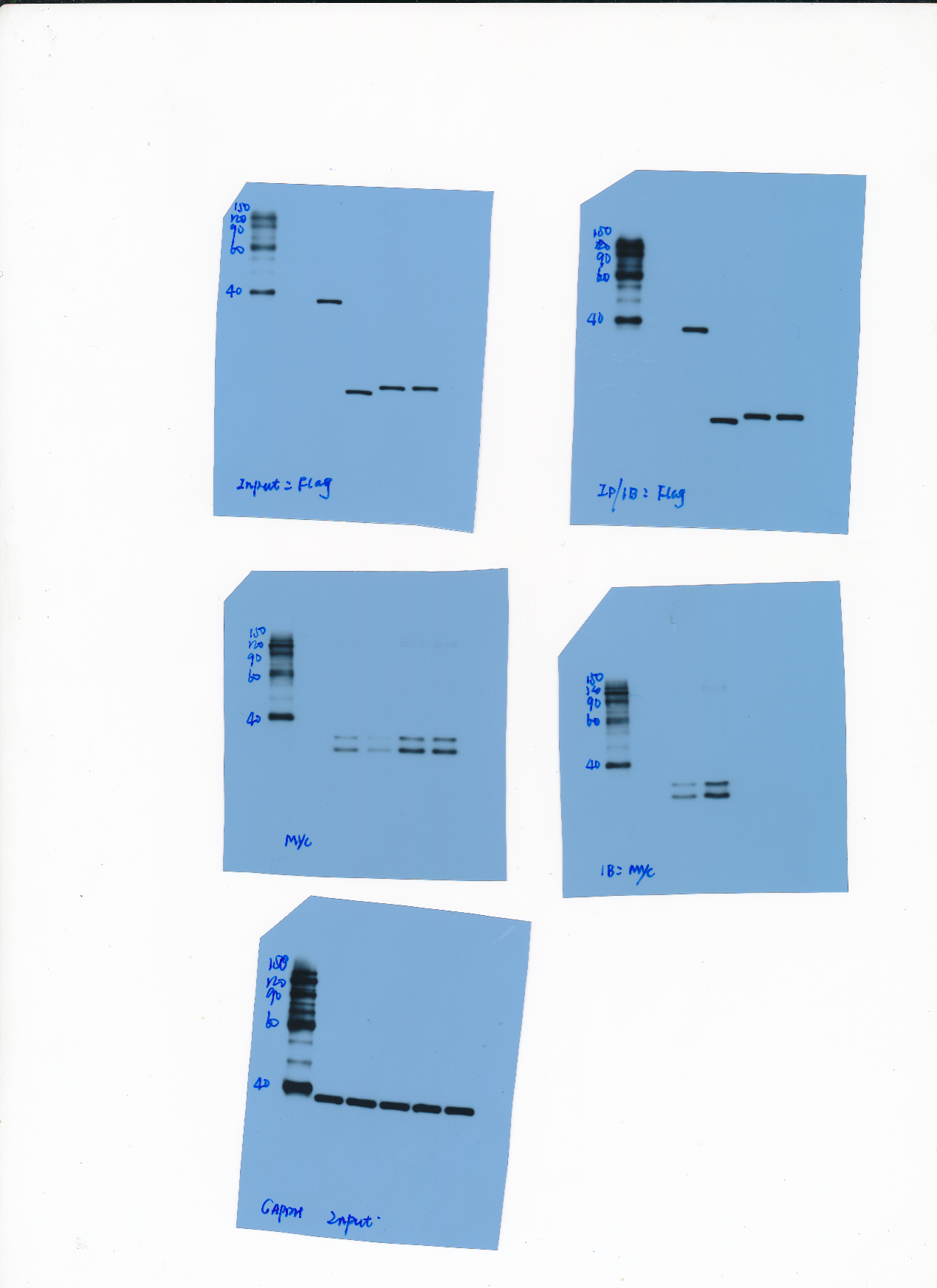


Fig 1I


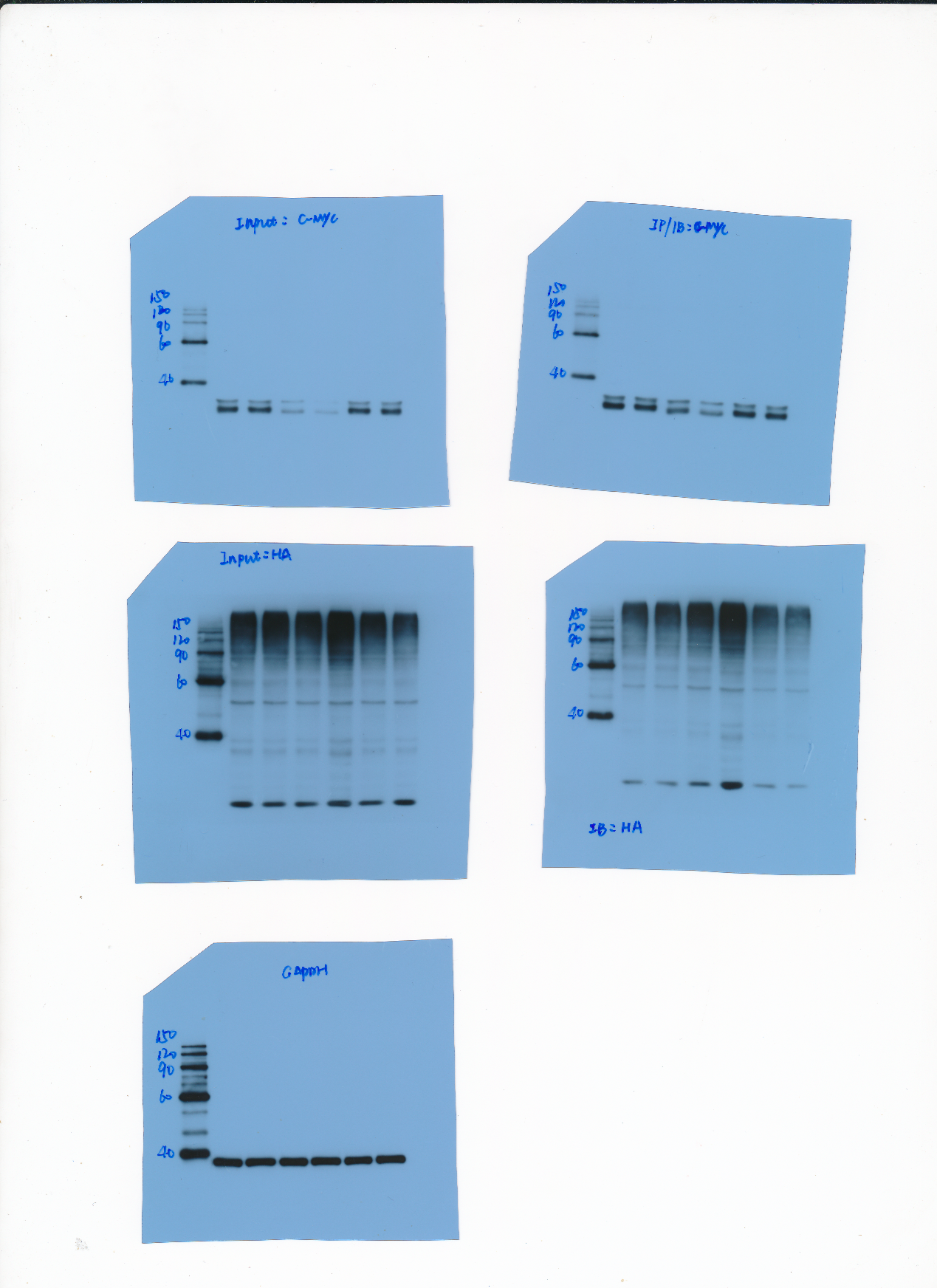


Fig 2A


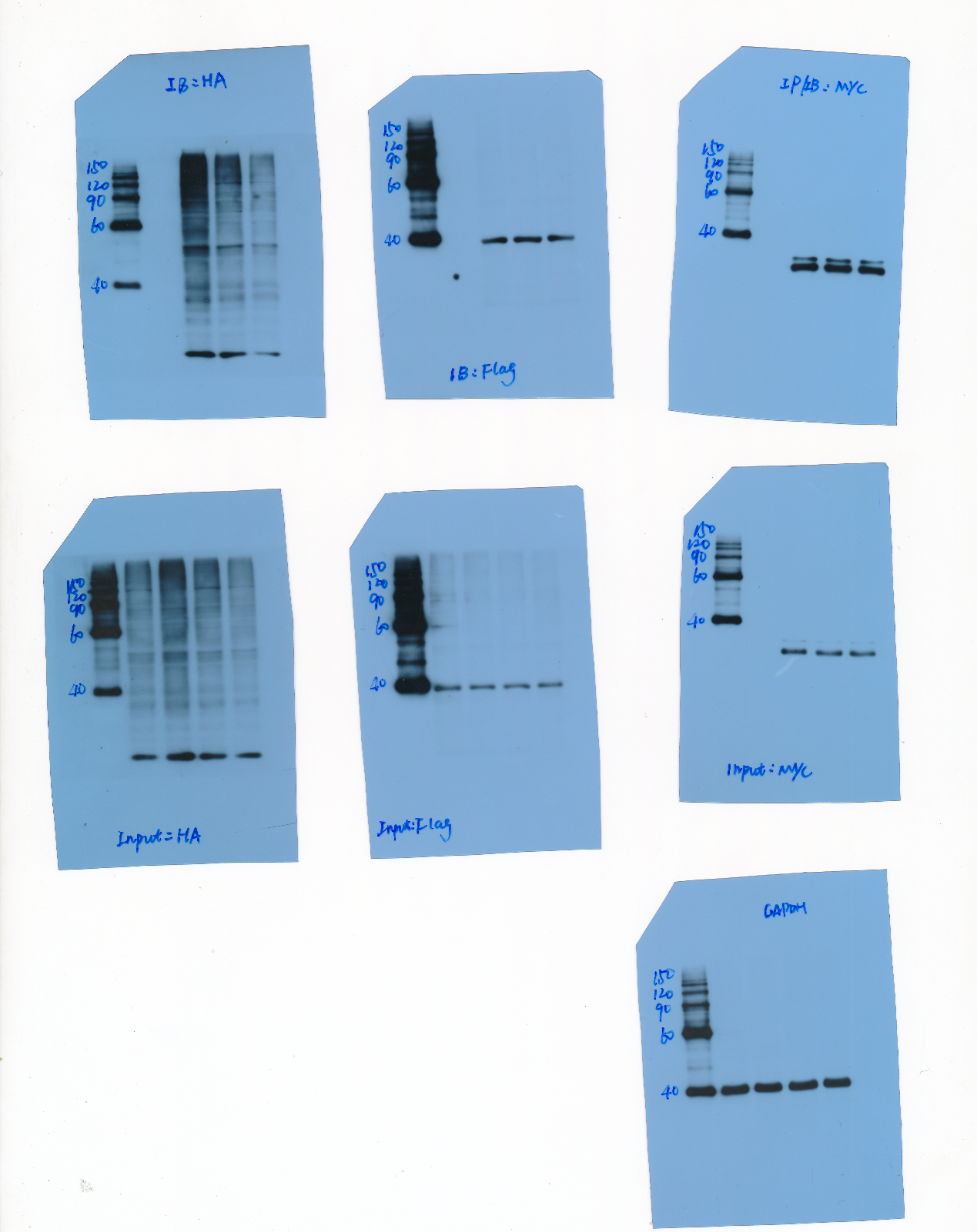


Fig 2B


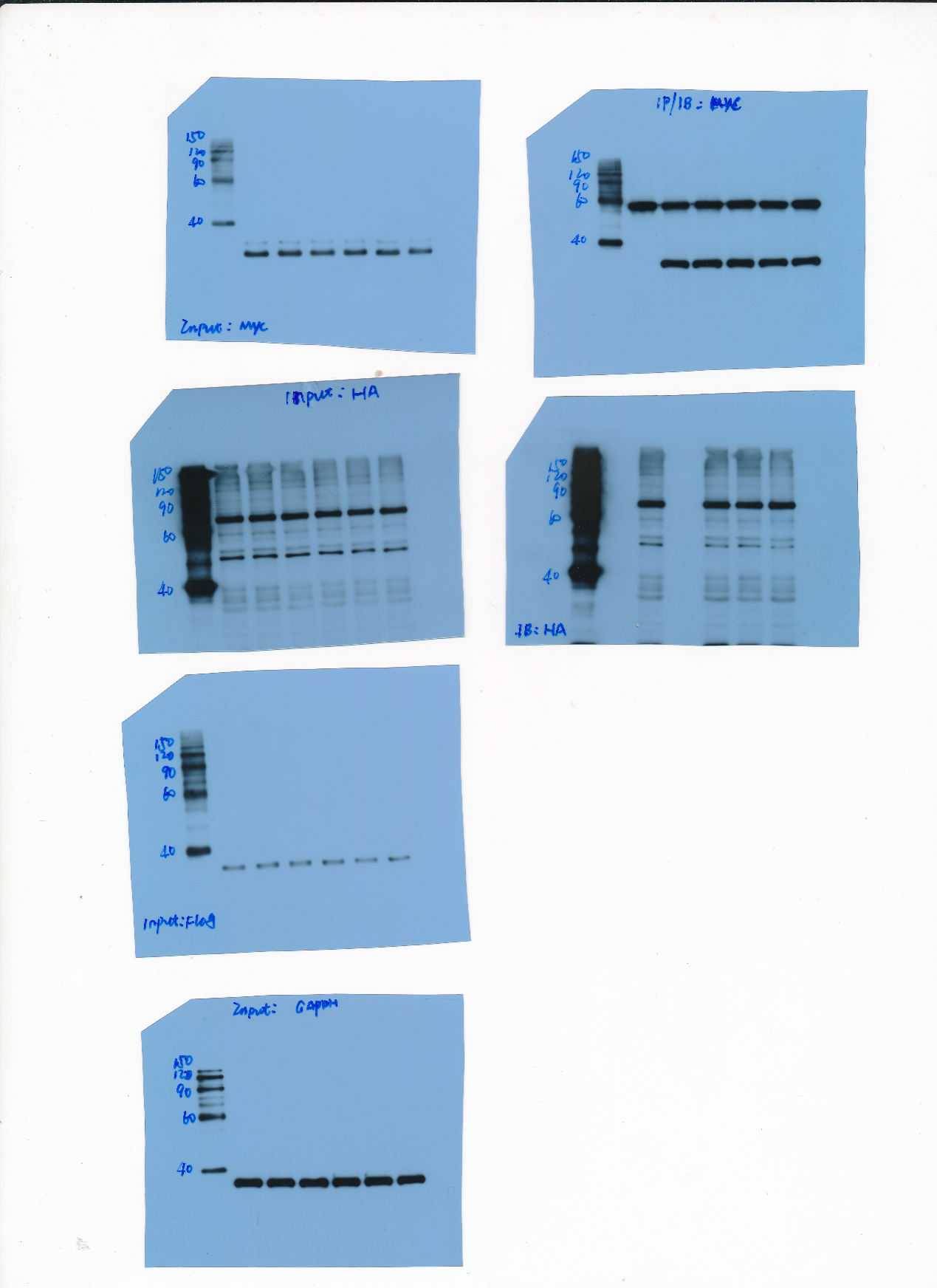


Fig 2C


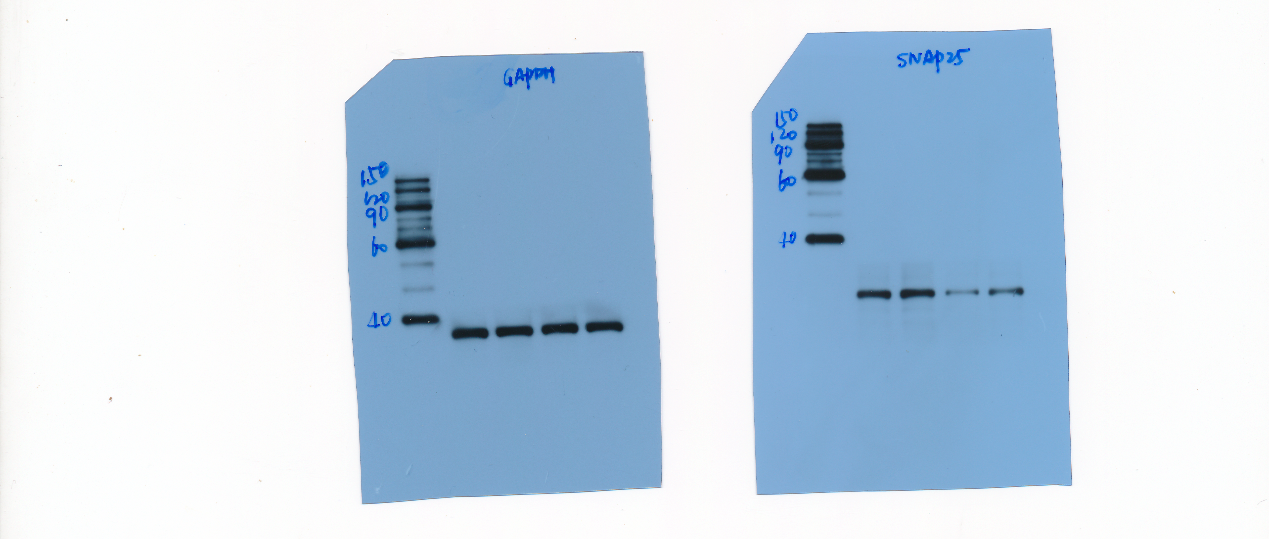


Fig 2D


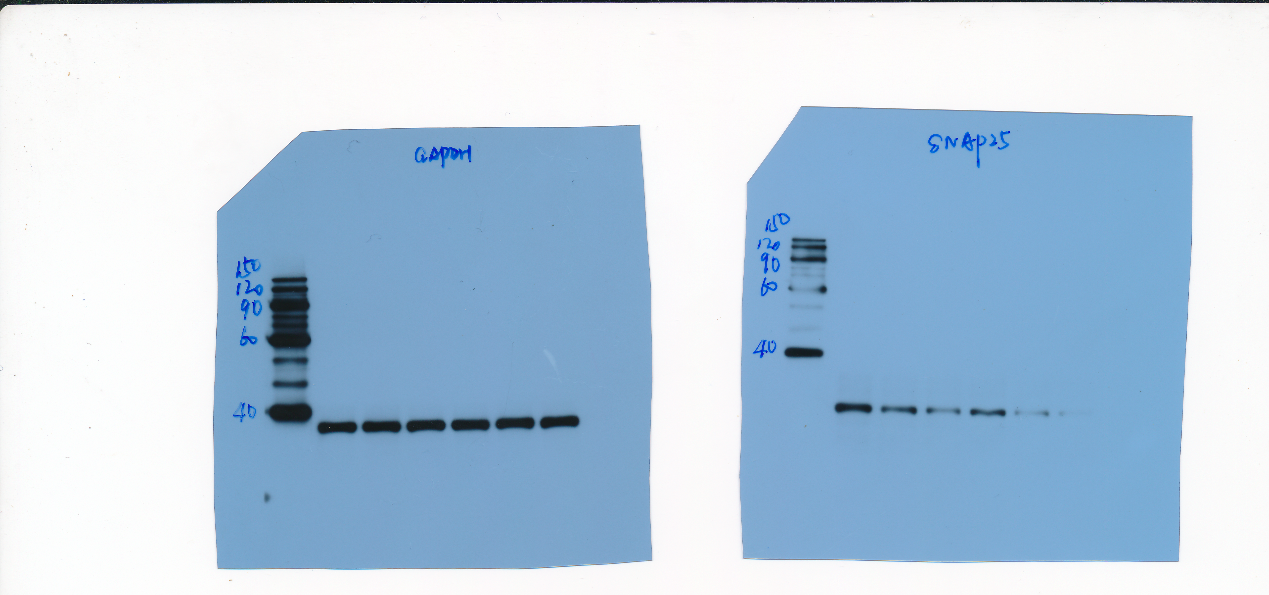


Fig 2F


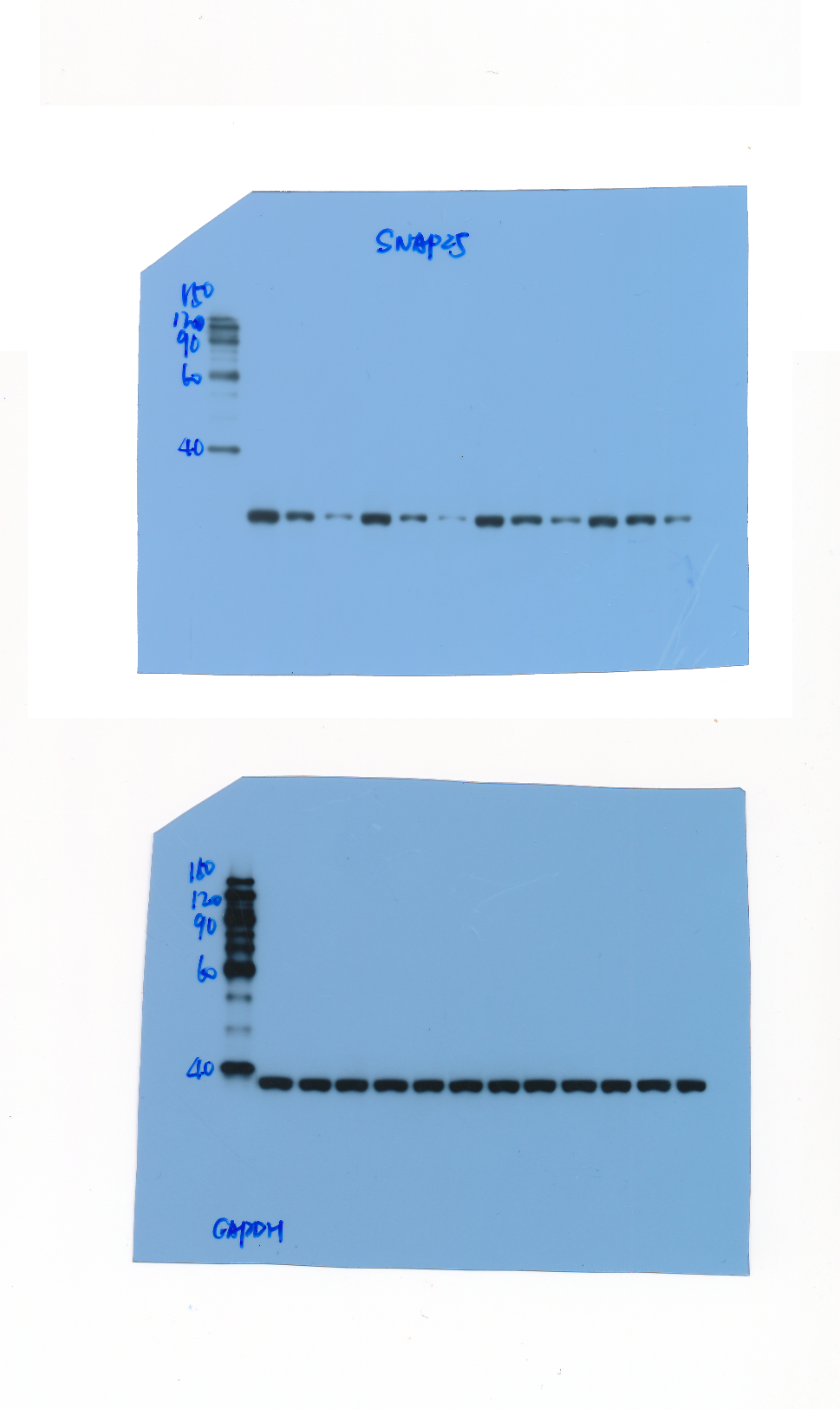


Fig 2H


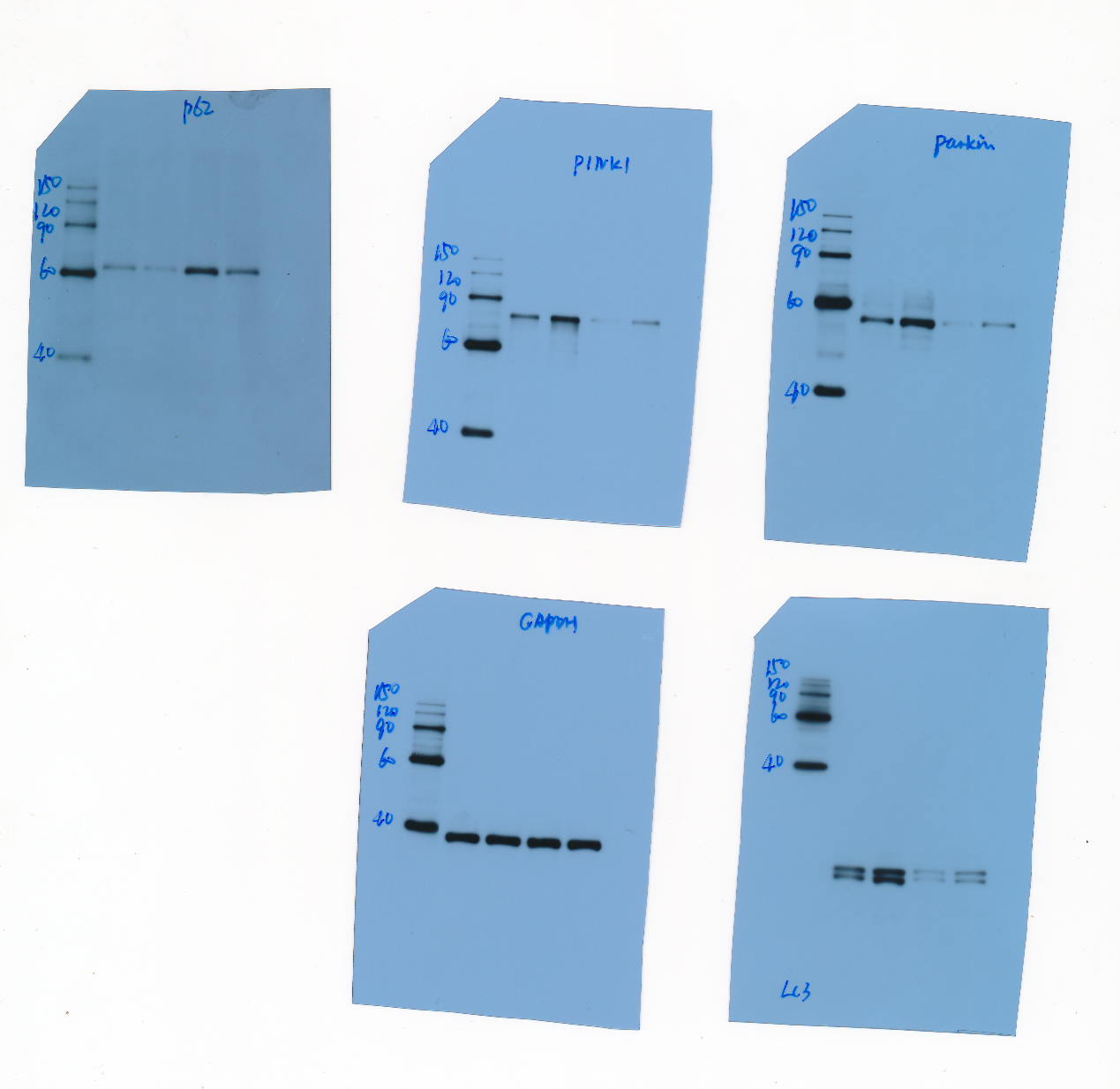


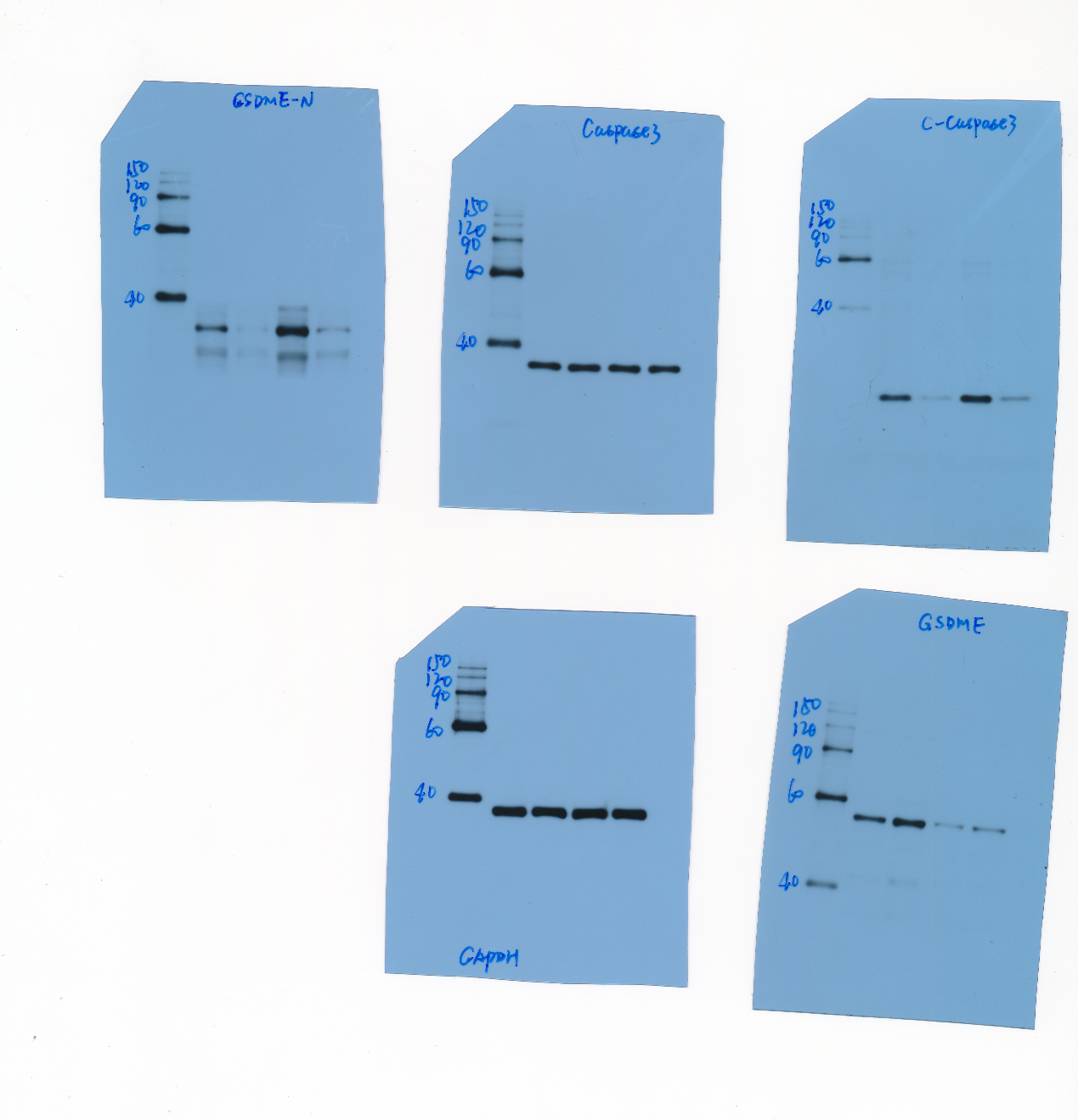

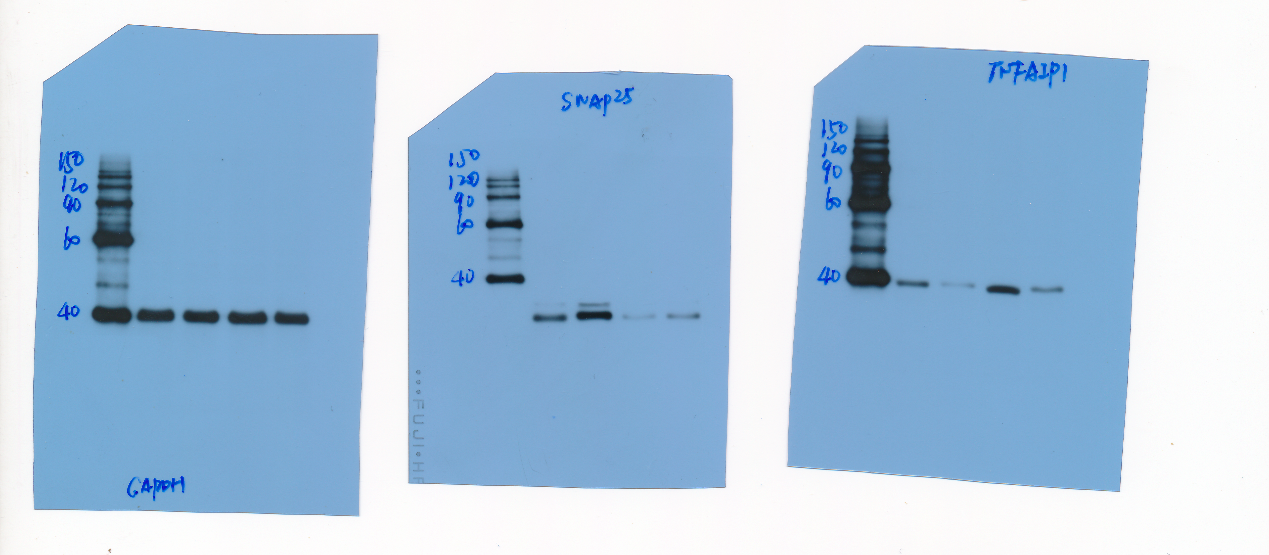


Fig 3D


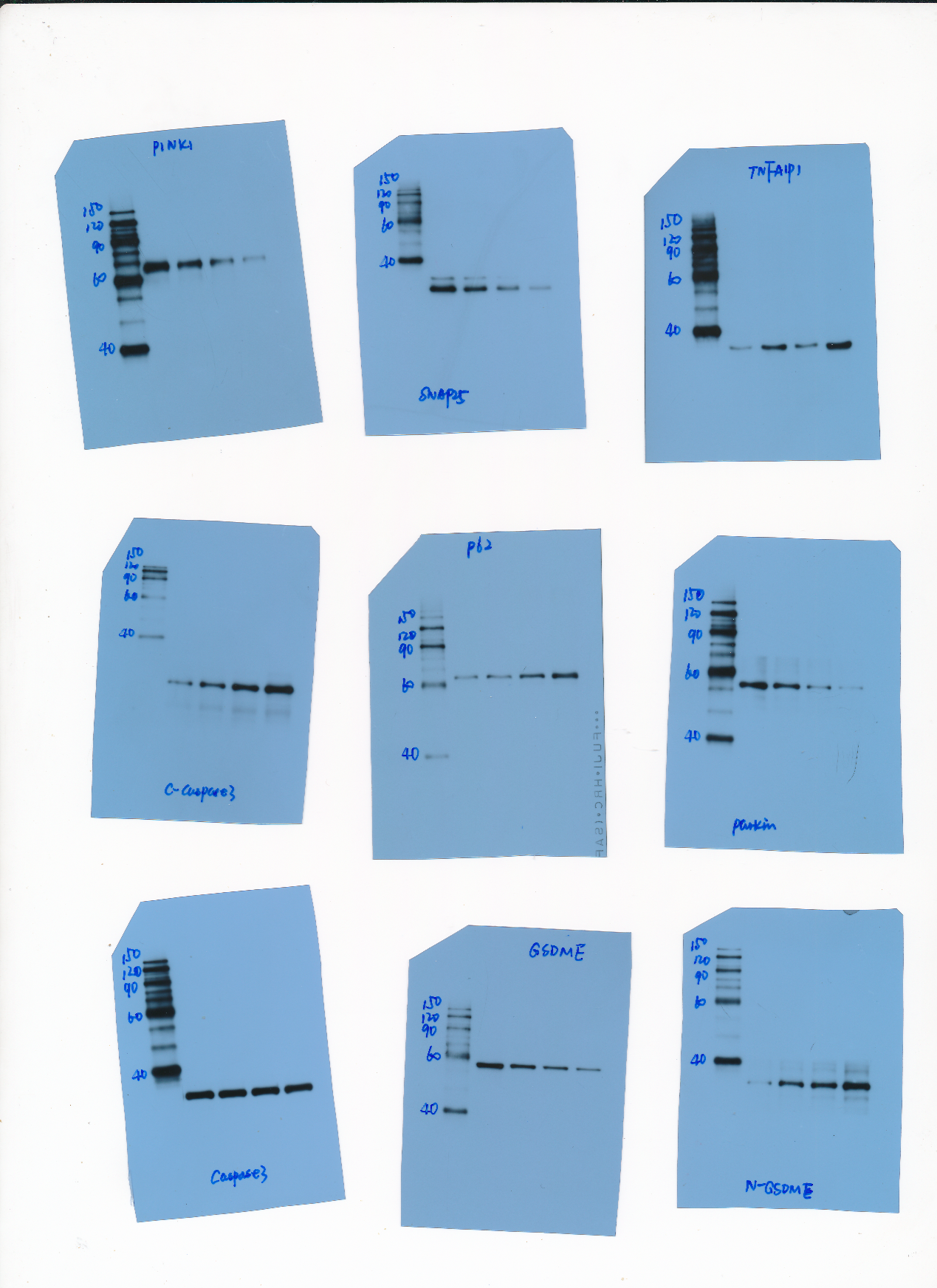

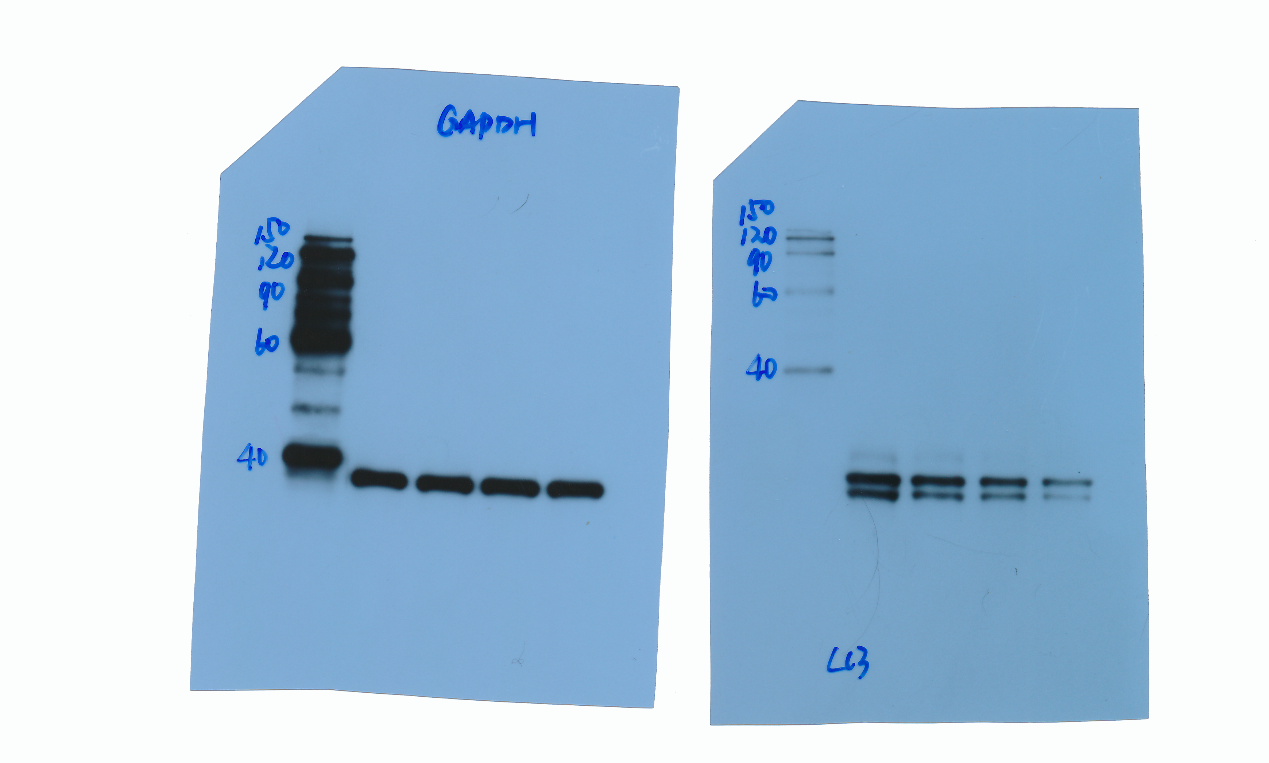


Fig 3H


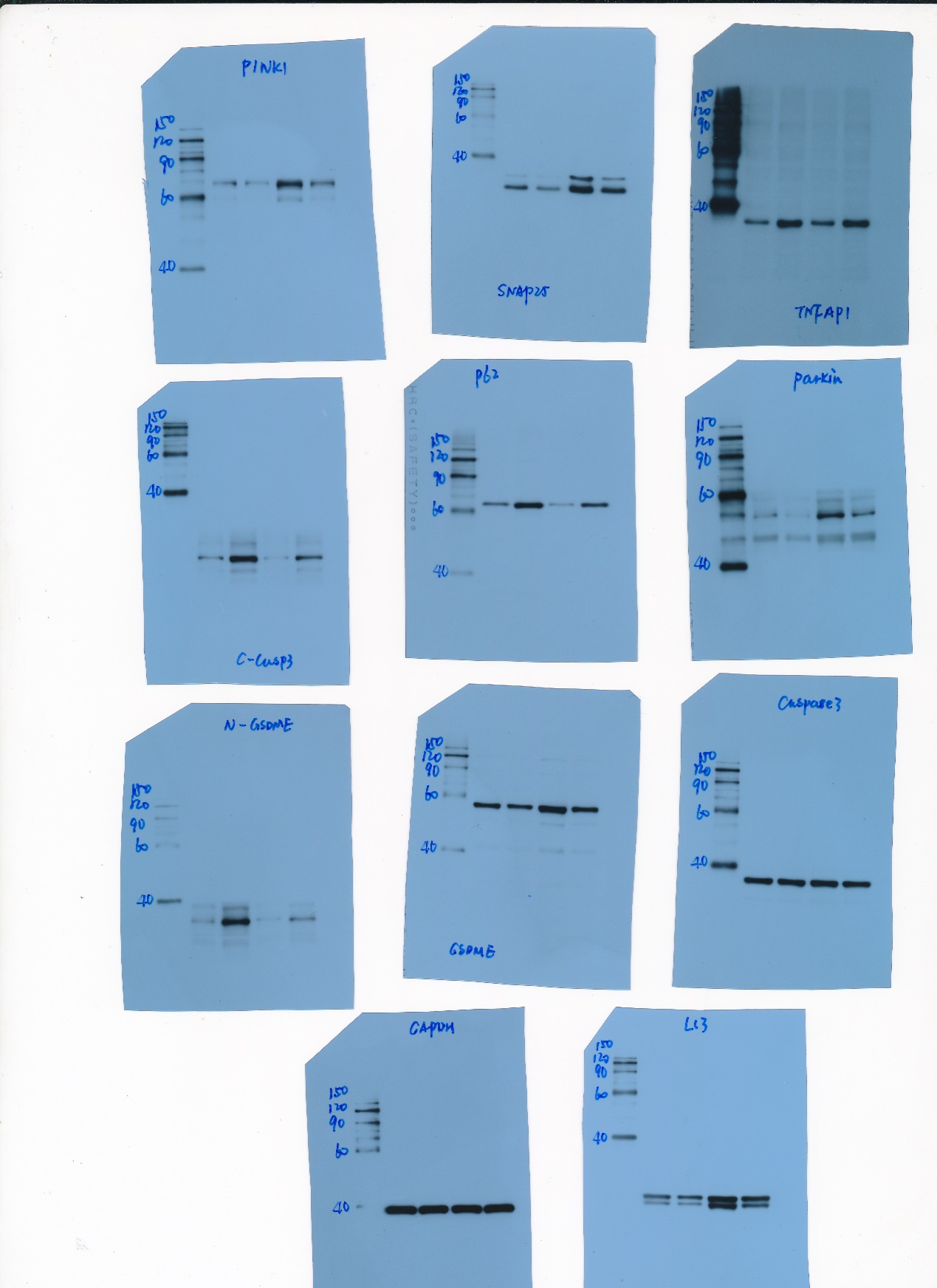


Fig 4D


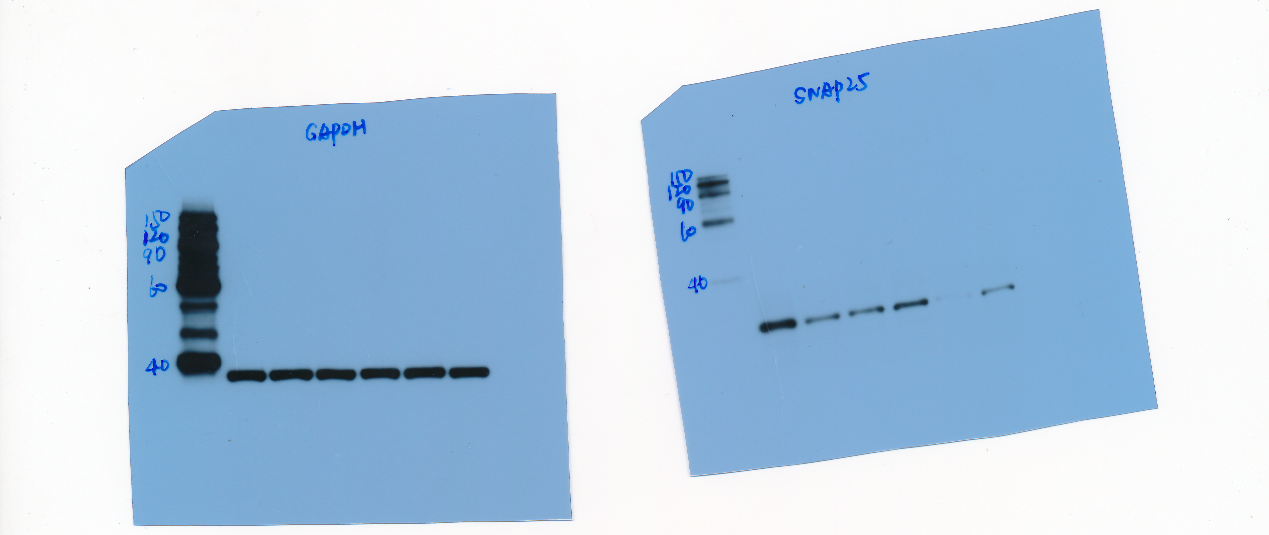

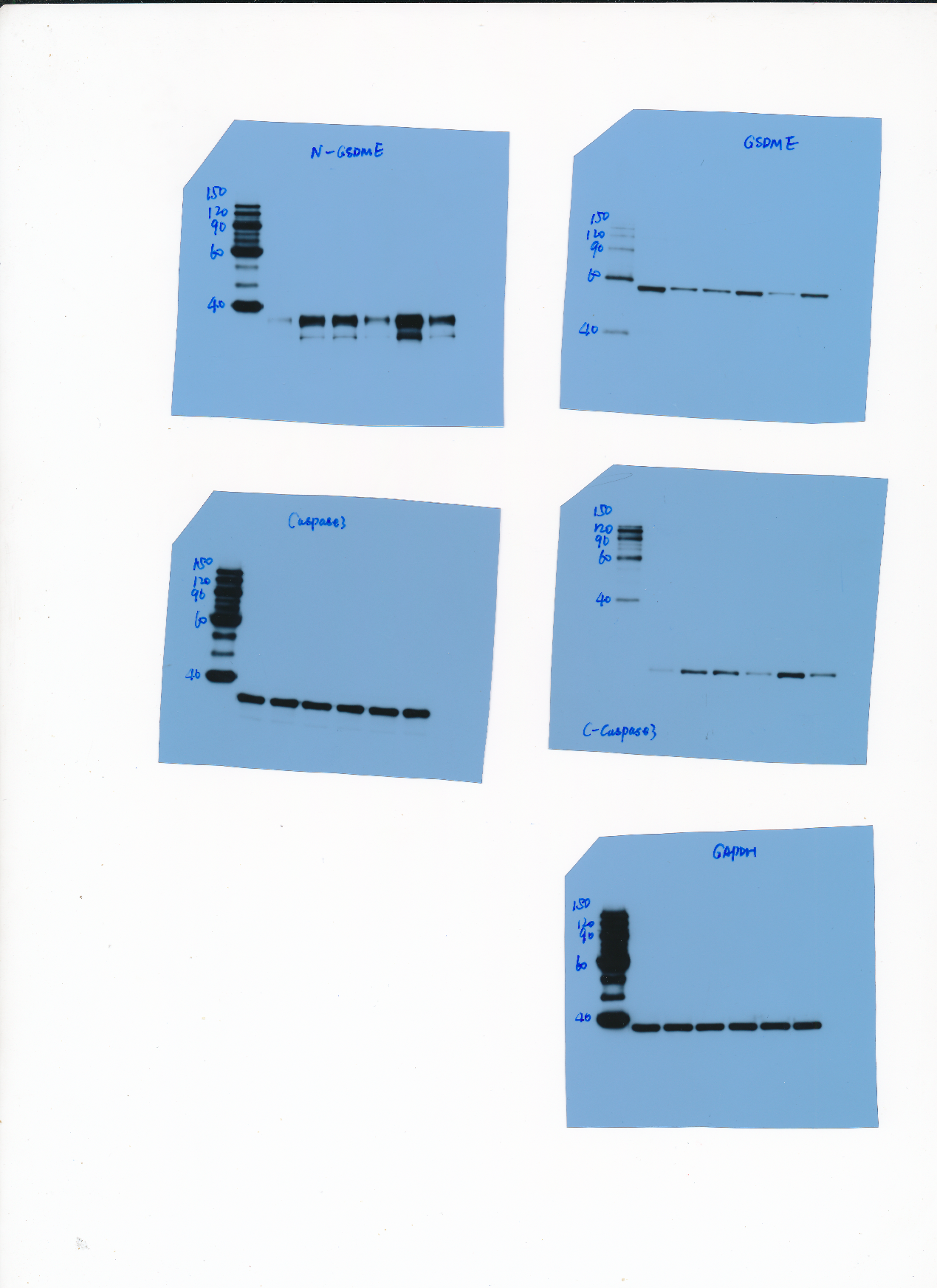

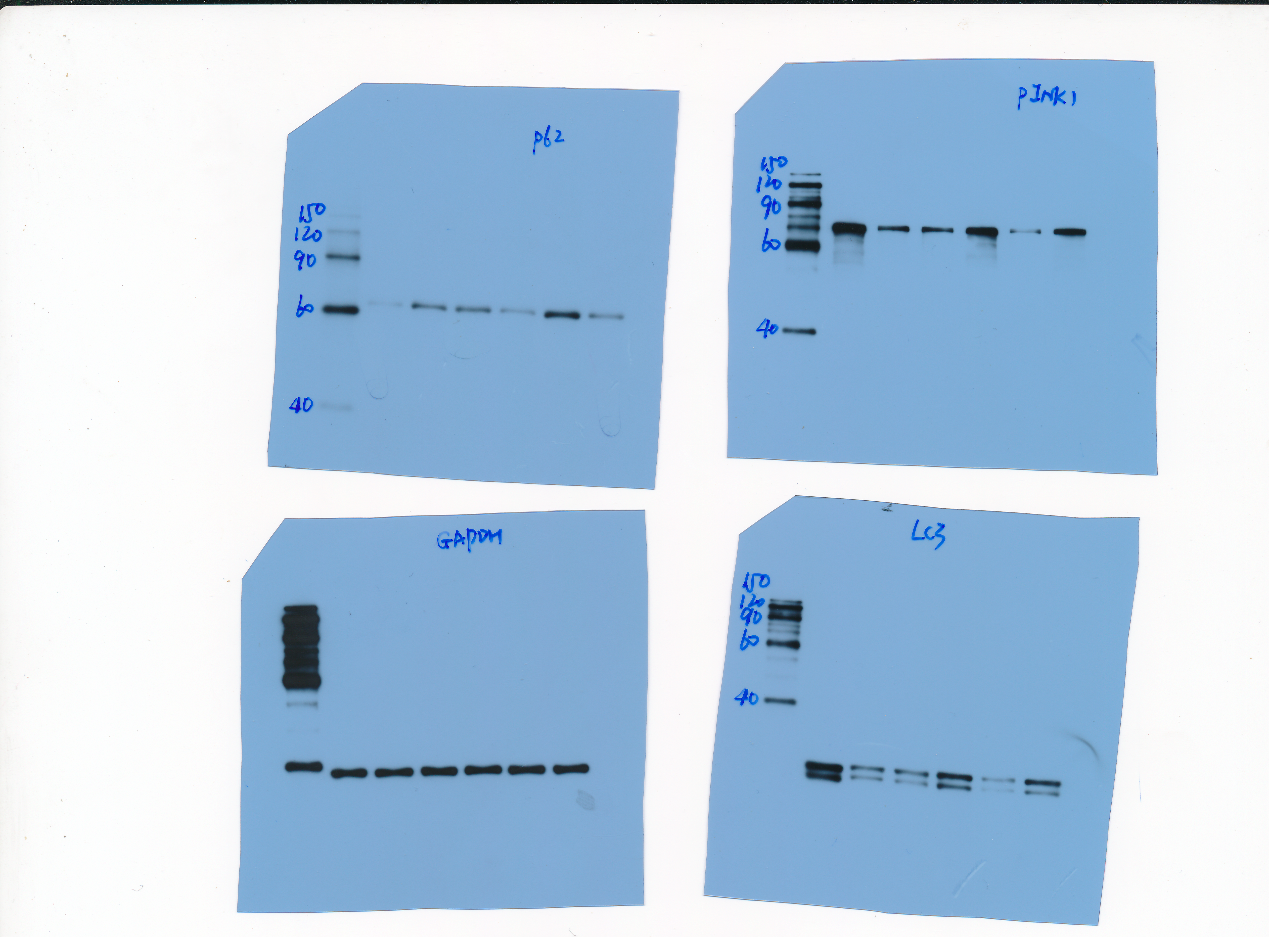

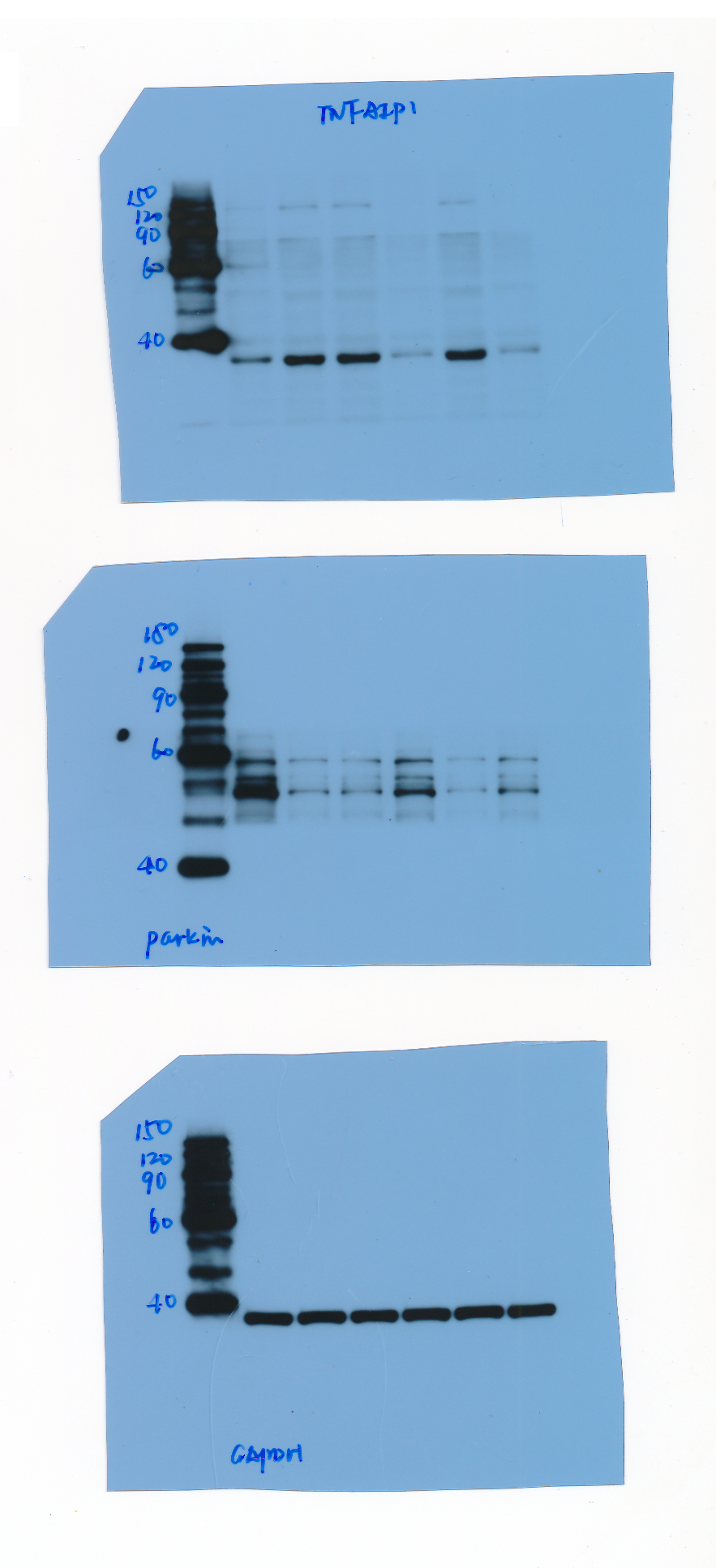


Fig 5F
